# Fungal Morphology, Iron Homeostasis, and Lipid Metabolism Regulated by a GATA Transcription Factor in *Blastomyces dermatitidis*

**DOI:** 10.1371/journal.ppat.1004959

**Published:** 2015-06-26

**Authors:** Amber J. Marty, Aimee T. Broman, Robert Zarnowski, Teigan G. Dwyer, Laura M. Bond, Anissa Lounes-Hadj Sahraoui, Joël Fontaine, James M. Ntambi, Sündüz Keleş, Christina Kendziorski, Gregory M. Gauthier

**Affiliations:** 1 Department of Medicine, University of Wisconsin, Madison, Madison, Wisconsin, United States of America; 2 Department of Biostatistics and Medical Informatics, University of Wisconsin, Madison, Madison, Wisconsin, United States of America; 3 Department of Biochemistry, University of Wisconsin, Madison, Madison, Wisconsin, United States of America; 4 Université du Littoral Côte d’Opale, Unité de Chimie Environnementale et Interactions sur le Vivant, Calais, France; 5 Department of Biochemistry, Department of Nutritional Sciences, University of Wisconsin, Madison, Madison, Wisconsin, United States of America; 6 Department of Statistics, University of Wisconsin, Madison, Madison, Wisconsin, United States of America; University of California, San Francisco, UNITED STATES

## Abstract

In response to temperature, *Blastomyces dermatitidis* converts between yeast and mold forms. Knowledge of the mechanism(s) underlying this response to temperature remains limited. In *B*. *dermatitidis*, we identified a GATA transcription factor, *SREB*, important for the transition to mold. Null mutants (SREBΔ) fail to fully complete the conversion to mold and cannot properly regulate siderophore biosynthesis. To capture the transcriptional response regulated by *SREB* early in the phase transition (0–48 hours), gene expression microarrays were used to compare *SREB*∆ to an isogenic wild type isolate. Analysis of the time course microarray data demonstrated *SREB* functioned as a transcriptional regulator at 37°C and 22°C. Bioinformatic and biochemical analyses indicated *SREB* was involved in diverse biological processes including iron homeostasis, biosynthesis of triacylglycerol and ergosterol, and lipid droplet formation. Integration of microarray data, bioinformatics, and chromatin immunoprecipitation identified a subset of genes directly bound and regulated by *SREB in vivo* in yeast (37°C) and during the phase transition to mold (22°C). This included genes involved with siderophore biosynthesis and uptake, iron homeostasis, and genes unrelated to iron assimilation. Functional analysis suggested that lipid droplets were actively metabolized during the phase transition and lipid metabolism may contribute to filamentous growth at 22°C. Chromatin immunoprecipitation, RNA interference, and overexpression analyses suggested that *SREB* was in a negative regulatory circuit with the bZIP transcription factor encoded by *HAPX*. Both *SREB* and *HAPX* affected morphogenesis at 22°C; however, large changes in transcript abundance by gene deletion for *SREB* or strong overexpression for *HAPX* were required to alter the phase transition.

## Author Summary

*Blastomyces dermatitidis* belongs to a group of human pathogenic fungi that convert between two forms, mold and yeast, in response to temperature. Growth as yeast (37°C) in tissue facilitates immune evasion, whereas growth as mold (22°C) promotes environmental survival, sexual reproduction, and generation of transmissible spores. Despite the importance of dimorphism, how fungi regulate temperature adaptation is poorly understood. We identified *SREB*, a transcription factor that regulates disparate processes including dimorphism. *SREB* null mutants, which lack *SREB*, fail to fully complete the conversion to mold at 22°C. The goal of our research was to characterize how *SREB* regulates transcription during the switch to mold. Gene expression microarray along with chromatin binding and biochemical analyses indicated that *SREB* affected several processes including iron homeostasis, lipid biosynthesis, and lipid droplet formation. *In vivo*, SREB directly bound and regulated genes involved with iron uptake, lipid biosynthesis, and transcription. Functional analysis suggested that lipid metabolism may influence filamentous growth at 22°C. In addition, *SREB* interacted with another transcription factor, *HAPX*.

## Introduction

*Blastomyces dermatitidis* belongs to a group of medically important ascomycetes that adapt to shifts in temperature by undergoing a morphologic switch known as the phase transition [1]. In soil (22°C), these pathogens grow as filamentous mold, which produce infectious conidia. Following soil disruption, aerosolized conidia and mold fragments inhaled into the lungs of a mammalian host (37°C) convert into pathogenic yeast [1,2]. In the yeast form, *B*. *dermatitidis* is able to evade host immune defenses to cause pneumonia and disseminate to other organs such as the bone or brain [3–5]. This adaptive response, which is essential for virulence [2], is conserved in *Histoplasma capsulatum*, *Coccidioides immitis*, *Coccidioides posadasii*, *Paracoccidioides brasiliensis*, *Penicillium marneffei*, and *Sporothrix schenckii* [1]. The transition in the opposite direction, yeast to mold, is postulated to facilitate survival outside the mammalian host, sexual reproduction by mating, and geographic dispersion through production of conidia [6].

The phase transition is a complex process that involves alteration of transcription, metabolism, lipid content, and cell wall carbohydrate composition [7–13]. In *B*. *dermatitidis* and *H*. *capsulatum*, *DRK1* (dimorphism-regulating kinase-1) and *RYP1-4* (required for yeast phase) promote the temperature-dependent conversion from mold to yeast, respectively [2,7,14,15]. The transition to yeast at 37°C is essential for virulence; deletion of *DRK1* renders *B*. *dermatitidis* and *H*. *capsulatum* avirulent during experimental murine pulmonary infection [2]. In *Penicillium marneffei*, *HGRA* (hyphal growth regulator) facilitates the conversion of yeast to hyphae at 25°C and *TUPA* promotes maintenance of mycelial phase morphology [16,17]. In *B*. *dermatitidis*, we discovered *SREB* (siderophore biosynthesis repressor in *Blastomyces*), which encodes a GATA transcription factor that affects the temperature-dependent morphologic switch and iron homeostasis [18]. *SREB* null (*SREB*∆) and insertional mutants fail to fully complete the conversion from yeast to sporulating mold after a drop in temperature from 37°C to 22°C, and under iron-replete conditions cannot repress the biosynthesis of iron-gathering siderophores [18]. The regulatory role of *SREB* on the phase transition appears conserved in the dimorphic fungi; knockdown of an *SREB* homolog in *H*. *capsulatum* led to failure of yeast cells to fully convert to mold at ambient temperature [19]. Deletion of *VMA1*, which encodes a catalytic subunit in a vacuolar ATPase involved with iron homeostasis and virulence, also impairs the conversion of *H*. *capsulatum* yeast to mold [20]. The kinetics of the temperature-dependent switch from yeast to mold is influenced by N-acetylglucosamine (GlcNAc), which is mediated by *NGT1* and *NGT2* transmembrane transporters [21]. Exogenous GlcNAc accelerated the conversion of *B*. *dermatitidis* and *H*. *capsulatum* yeast to mold at room temperature [21]. Although several genes have been identified to govern the morphologic switch, how these genes coordinate proper adaptation to temperature, which is reflected by growth as yeast at 37°C or mold at 22–25°C remains poorly understood.

GATA transcription factors bind DNA via zinc finger motifs to induce or repress transcription in response to environmental stimuli. In fungi, they regulate adaptive responses to light, temperature, nitrogen, and iron [22–28]. Moreover, GATA transcription factors are capable of governing disparate functions. *ASH1* in *Saccharomyces cerevisiae* inhibits mating type switching and under nitrogen poor conditions induces pseudohyphal growth [22,23]. *Cryptococcus neoformans CIR1*, which is a homolog of *SREB*, regulates genes important for thermotolerance at 37°C, capsule formation, iron uptake, and mating [24,25]

The goals of this study were to characterize the *SREB* regulon on a genome-wide scale using gene expression microarrays, identify a subset of genes directly bound and regulated by *SREB in vivo*, and functionally test the impact of *SREB*-regulated genes on the phase transition. Because the morphologic defect in *SREB*∆ occurred within 24–48 hours of a drop in temperature, we focused our attention to the early part of the phase transition where *B*. *dermatitidis* yeast adapt to 22°C and begin the conversion to mold. The data generated from our approach indicated that the GATA transcription factor encoded by *SREB* affects transcription at 37°C and 22°C, and binds to genes involved with diverse (iron and non-iron) processes. In addition to functioning as a major regulator of iron assimilation, *SREB* affected the biosynthesis of lipids at 37°C and 22°C, and lipid droplet formation at 22°C. Similar to filamentous fungi such as *Aspergillus fumigatus*, *SREB* was in a regulatory circuit with the bZIP transcription factor *HAPX*. Deletion of *SREB* or overexpression of *HAPX* resulted in a defect in the morphologic switch at 22°C.

## Results

### The morphologic defect in *SREB*∆ occurs early in the phase transition

**Fig 1:**
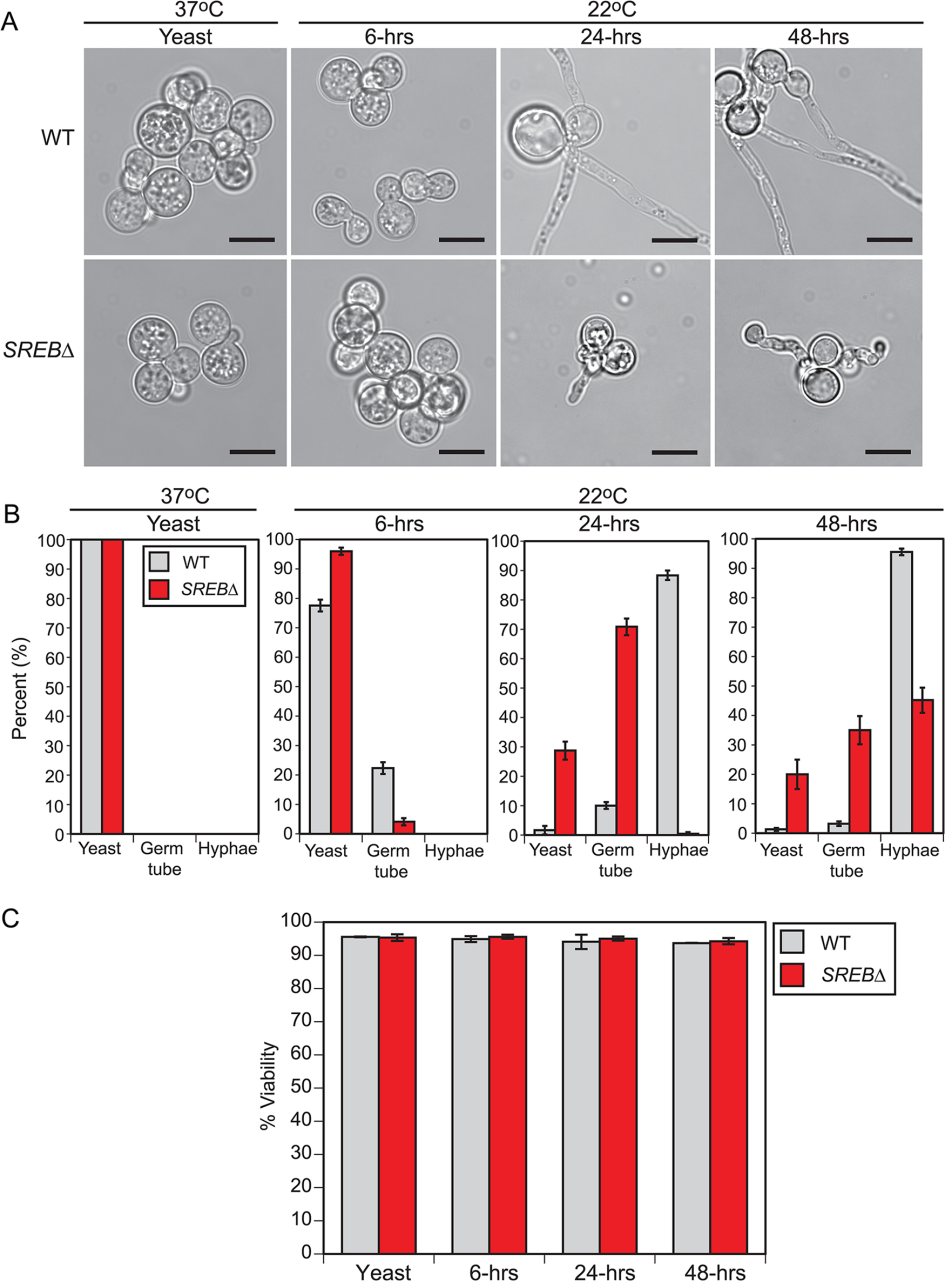
Cell morphology during the early stages following a drop in temperature. **(A)** Morphology of wild-type (WT) and *SREB*∆ at 37°C and at 6, 24, and 48-hrs after a drop in temperature at 22°C. When compared to WT, *SREB*∆ cells exhibited a delay in the filamentous growth (germ tube and hyphal formation). At 22°C, *SREB*∆ filaments were abnormal in morphology. Scale bar equals 10 μm. **(B)** Percentage of WT and *SREB*∆ cells with yeast morphology, germ tube development, and hyphal growth at 37°C and 22°C. Results were averaged from 2 independent experiments (> 200 cells counted in duplicate). **(C)** Percent of WT and *SREB*∆ cells that are viable at 37°C and 22°C. Results were averaged from 2 independent experiments (> 200 cells counted in duplicate)

To investigate the kinetics of the phase transition defect in *SREB*∆, we compared it to an isogenic wild-type (WT) control at 37°C and at 6, 24, and 48-hrs following a drop in temperature to 22°C (**Fig 1A–1C**). At 37°C, WT and *SREB*∆ grew as budding yeast (**Fig 1A and 1B**). After 6-hrs at 22°C, WT and *SREB*∆ continued to have a yeast morphology with 22% and 4% cells exhibiting early germ tube development, respectively (**Fig 1A and 1B**). At 24 and 48-hrs at 22°C, the germ tubes of WT cells had elongated to become hyphae (**Fig 1A and 1B**). In contrast, sharp morphologic differences between *SREB*∆ and WT became apparent at 24 and 48-hrs at 22°C with *SREB*∆ demonstrating a delay in germ tube formation and stunted, misshapen germ tubes and hyphae (**Fig 1A and 1B**). Deletion of *SREB* did not affect cell viability (**Fig 1C).**

On the basis of the time-course data, we postulated that transcriptional alterations responsible for the morphologic defect in *SREB*∆ occurred early in the phase transition (conversion of wild-type *B*. *dermatitidis* to sporulating mold requires 10–14 days incubation at 22°C). To optimize detection of transcriptional events associated with the phase transition (e.g., germ tube formation and elongation), we focused on the first 48-hrs at 22°C, which was when the morphologic defect in *SREB*∆ first became apparent. Microarray analyses of later time points (e.g., 7 or 14 days at 22°C) were not performed because the transcriptional differences between *SREB*∆ and WT would reflect differences in filamentous growth rather than the morphologic switch. We harvested RNA from 3 biological replicates for WT and *SREB*∆ at 37°C, and 6, 24, and 48-hrs following a drop in temperature to 22°C. The 37°C time point served a baseline, whereas the 22°C time points provided insight into transcription at the beginning of germ tube formation (6-hrs) and during the transition from germ tubes to hyphae (24-hrs and 48-hrs) (**Fig 1A and 1B**). All cultures were grown under iron-replete conditions (10 μM FeSO_4_) to optimize transcription of *SREB* in the isogenic WT isolate. Iron induces transcription of *SREB* and is required for the binding of SREB homologs, such as *H*. *capsulatum* SRE1, to DNA [18,29].

LIMMA and EBarrays statistical packages were used to identify differentially expressed genes in *SREB*∆ versus WT isolates at each time point, [30,31]. Two statistical methods were used because each makes slightly different assumptions, and we wanted to ensure that our results were robust to the statistical method used. Both methods identified differentially expressed (DE) genes at all time points (**Table 1**and **S1 Table**). All DE genes identified by EBarrays were also DE by LIMMA and, consequently, to be conservative, subsequent analyses herein include genes that were classified as DE by EBarrays (**S1 Table**). The majority of DE genes in *SREB*∆ exhibited a 1.5-fold or greater change in transcript abundance when controlling the false discovery rate at 5% (**Table 1**). In the yeast phase (37°C), the ratio of DE genes with increased to decreased transcription was 1.56:1. At 22°C, this ratio was nearly equal (1.16:1 at 6-hrs, 0.87:1 at 24-hrs; 1.02:1 at 48-hrs). At 22°C, the majority of DE genes (570 / 1109 for 6-hrs, 1196 / 1772 for 24-hrs, 1345 / 1910 for 48-hrs) did not overlap with DE genes found at baseline (37°C). Thus, differential expression of these genes was unique to the 22°C time points. Collectively, these data indicate that deletion of *SREB* affected transcription at 37°C and 22°C.

**Table 1:** Differentially expressed (DE) genes in *SREB*∆ versus wild type at 37°C and 22°C.

		Fold change of DE genes				
	DE Genes	≥1.5	≥2.0	≥3.0	≥4.0	≥5.0
Yeast 37°C	1,410	1,403 (99.5%)	912 (64.7%)	315 (22.3%)	156 (11.1%)	112 (7.9%)
↑Transcription	859	853	550	182	79	55
↓Transcription	551	550	362	133	77	57
6-hours 22°C	1,109	1,109 (100%)	678 (61.1%)	217 (19.6%)	100 (9.0%)	69 (6.2%)
↑Transcription	596	596	375	124	61	41
↓Transcription	513	513	303	93	39	28
24-hours 22°C	1,772	1,755 (99.0%)	1108 (62.5%)	417 (23.5%)	203 (11.5%)	104 (5.9%)
↑Transcription	823	815	513	207	101	55
↓Transcription	949	940	595	210	102	49
48-hours 22°C	1,910	1,869 (97.9%)	1,076 (56.3%)	403 (21.1%)	203 (10.6%)	115 (6.0%)
↑Transcription	963	939	537	217	118	70
↓Transcription	947	930	539	186	85	45

DE Genes refers to genes determined to be DE by both LIMMA and EBarrays analyses (FDR controlled at 5%)

Fold change values reflect median fold change between *SREB*∆ and wild-type and includes genes with increased and decreased transcription. Fold change values of 1.5, 2.0, 3.0, 4.0, and 5.0 correspond to log_2_ fold change of 0.585, 1.000, 1.585, 2.000, and 2.322 in S1 Table, respectively.

### Deletion of *SREB* affected the transcription of genes involved with diverse processes

To facilitate functional classification of DE genes, gene ontology (GO) terms were assigned to proteins encoded by the *B*. *dermatitidis* genome using InterProScan. GO analysis revealed DE genes in *SREB*∆ were enriched for 17 GO terms and the number of enriched GO terms increased across the time course, which likely reflected the sharp morphologic differences between *SREB*∆ and WT at 22°C (**Table 2; S2 Table,** and **S3 Table**). Catalytic activity (GO:0003824), metabolic process (GO:0008152), oxidoreductase activity (GO:0016491), and oxidation-reduction process (GO:0055114) contained the largest number of DE genes that were enriched across all 22°C time points (**Table 2, S3 Table,** and **S1 Fig**). To assess potential function for genes within GO categories, we integrated GO term, TBLASTN (PubMed and *Saccharomyces cerevisiae* databases), and PFAM analyses (**S2 Table**). The predicted functions these genes were diverse (**S2 Table**) and many DE genes were shared among the enriched gene ontologies (**S2 Table** and **S4 Table**). Integrative analysis suggested several biological themes including iron ion binding, lipid biosynthesis, and amino acid metabolism.

**Table 2:** Gene ontology (GO) enrichment analysis.

GO Term	GO ID	37°C	22°C		
		Yeast	6-hrs	24-hrs	48-hrs
**Ontology: Molecular Function**					
Iron Ion Binding	GO:0005506	X (0.019)	—	X (0.002)	—
Catalytic Activity	GO:0003824	—	X (< 0.001)	X (< 0.001)	X (< 0.001)
Oxidoreductase Activity	GO:0016491	—	X (0.006)	X (< 0.001)	X (< 0.001)
FAD binding	GO:0050660	—	X (0.092)	—	X (0.093)
Amino Acid Transmembrane Transporter Activity	GO:0015171	—	—	X (0.018)	X (0.097)
Hydrolase Activity, hydrolyzing O-glycosyl compounds	GO:0004553	—	—	X (0.048)	X (0.051)
Transporter Activity	GO:0005215	—	—	X (0.057)	X (0.079)
**Ontology: Biologic Process**					
Transmembrane Transport	GO:0055085	X (0.092)	—	X (< 0.001)	X (< 0.001)
Metabolic Process	GO:0008152	—	X (< 0.001)	X (< 0.001)	X (< 0.001)
Oxidation-Reduction Process	GO:0055114	—	X (0.092)	X (< 0.001)	X (< 0.001)
Fatty Acid Biosynthetic Process	GO:0006633	—	—	X (0.018)	—
Amino Acid Transport	GO:0006865	—	—	X (0.018)	X (0.097)
Transport	GO:0006810	—	—	—	X (0.008)
Carbohydrate Metabolic Process	GO:0005975	—	—	—	X (0.097)
**Ontology: Cellular Component**					
Integral to Membrane	GO:0016021	X (0.092)	X (0.006)	—	X (0.011)
Membrane	GO:0016020	—	—	X (0.028)	X (0.006)
Endoplasmic Reticulum	GO:0005783	—	—	X (0.043)	—

X indicates enrichment. Numbers in parentheses are *q*-values. “—” indicates no enrichment. GO ID refers to gene ontology identification number.

Differentially expressed genes predicted to encode iron-binding proteins included those with electron-carrier activity and stress response. The majority of DE genes encoding proteins with electron carrier functions contained cytochrome (19 / 48 genes, 39.6%), acyl-CoA dehydrogenase/oxidase (11 / 48, 22.3%), and ferric reductase (4 / 48 genes, 8.3%) domains. Cytochrome and acyl-CoA dehydrogenase/oxidase encoding genes exhibited reduced transcription (22 / 30 genes, 73.3%) and known functions were limited to sterol biosynthesis (BDBG_01678, BDBG_03201, BDBG_04743, BDBG_06540) and beta-oxidation (BDBG_02355, BDBG_00463), respectively. Transcription of cytochromes involved with oxidative phosphorylation were unaffected by deletion of *SREB*∆ (BDBG_01291, BDBG_09317, BDBG_06453, BDBG_08893, BDBG_05852, BDBG_07205, BDBG_04875, 08630, BDBG_03418, BDBG_04645, BDBG_05869, BDBG_01464, BDBG_05537, BDBG_06649). Genes encoding proteins with ferric reductase domains had increased transcription in *SREB*∆ (3 / 4 genes, 75%) including an NADPH oxidase (BDBG_06745), a metalloreductase (BDBG_07462), and a FRE family reductase (BDBG_09322). Genes involved with stress response included those encoding peroxidases and catalases. Four peroxidases were DE including 3 with unknown function and 1 with a haem peroxidase domain. Similar to *H*. capsulatum, *B*. *dermatitidis* encodes 3 catalase isoforms (A, B, and P). In *SREB*∆, these isoenzymes (BDBG_05307, BDBG_08680, BDBG_07107) are DE at select time points and not throughout the time course (e.g., 0.75 and 1.38 log_2_ increase for catalase A at 24 and 48-hrs 22°C; 1.06 and 1.24 log_2_ increase for catalase B at 37°C and 48-hrs 22°C; log_2_ 1.21 and 0.62 decrease for catalase P at 24 and 48-hrs 22°C). Similarly, superoxide dismutase was DE only at 48-hrs 22°C (BDBG_07234; log_2_ 0.68 decrease at 48-hrs).

Deletion of *SREB* decreased transcription of genes in the triacylglycerol (TAG) and ergosterol biosynthetic pathways, but had limited impact on genes involved with phospholipid biosynthesis.

In *SREB*∆, 57.1% (8 / 14) of genes in the triacylglycerol (TAG) biosynthetic pathway and 66.7% (14 / 21) of genes in the ergosterol biosynthetic pathways were DE**.** The majority of these DE genes (7 of 8 for glycerolipid pathway, 12 of 14 for ergosterol pathway) had reduced transcript abundance in *SREB*∆ versus WT. In contrast, deletion of *SREB* had limited affect on phosopholipid biosynthesis in the Kennedy and cytidine diphosphate diacylglycerol (CDP-DAG) pathways (**S2 Fig**). In the Kennedy pathway, two choline transporters and two biosynthetic genes for phosphatidylcholine (PC) were DE in *SREB*∆, but the impact on transcription was divergent (**S2 Fig**). Transcription of genes for phosphadidylserine (PS), phosphatidylethanolamine (PE), and phosphatidylinositol (PI) biosynthesis were not altered. Moreover, homologs of *S*. *cerevisiae OPI3* and *PIS1*, which are involved with conversion of PE to PC had similar transcript abundance in *SREB*∆ and WT (**S2 Fig**). The only genes with altered transcription in the CPD-DAG pathway were involved in the biosynthesis of cardiolipin. These two genes, which were homologs of *S*. *cerevisiae CRD1* (BDBG_07720) and *PGS1* (BDBG_03699) had 0.77 and 0.95 log_2_ decrease in transcription in *SREB*∆ (versus WT) at 24 and 48-hrs 22°C, respectively (**S2 Fig**). Homologs for *S*. *cerevisiae INO2*, *INO4*, and *OPI1*, which are transcription factors that regulate phospholipid biosynthesis [32], were not identified in the *B*. *dermatitidis* genome by tBLASTn analysis.

*SREB*∆ cells had reduced transcription for genes in 16 of 21 amino acid biosynthetic pathways at 24 and 48-hrs 22°C (**S3 Fig**). For example, nearly all genes (12 / 14, 85.7%) involved with branched chain amino acid biosynthesis (i.e., valine, leucine, isoleucine) had decreased transcript abundance (**S3 Fig**). Biosynthetic genes for methionine, glutamate, and serine were not altered by deletion of *SREB*∆ at 24 or 48-hrs 22°C, whereas cysteine and asparagine biosynthetic genes had increased transcription (**S3 Fig**). At 37°C, deletion of *SREB* had limited effect on biosynthetic genes; however, an amino acid acetyltransferase (BDBG_00643) involved with first step in ornithine biosynthesis had a 1.1 log_2_ increase (**S3 Fig**); ornithine serves as a substrate for siderophore biosynthesis.

Transcript abundance for DE genes encoding amino acid transporters was also altered at 24-hrs 22°C (14 / 20 genes) and 48-hrs 22°C (13 / 20 genes). At these time points, transcript abundance for transporters was decreased for proline (BDBG_04825), increased for cationic amino acids (BDBG_03808, BDBG_08628) and **γ**-aminobutyrate (BDBG_03404), and heterogenous for methionine/cysteine (BDBG_00563, BDBG_03572, BDBG_00074) as well as for transporters with unknown function (BDBG_00367, BDBG_02461, BDBG_03769, BDBG_06532, BDBG_07277, BDBG_09387). In addition, 2 putative mitochondrial transporters, BDBG_04548, BDBG_05901, had decreased transcription. Of the predicted amino acid transporters, BDBG_08000, which is a homolog of *S*. *cerevisiae GAP1* (general amino acid permease), had the largest change in transcript abundance with log_2_ 1.76–2.65 increase at 22°C in *SREB*∆ (versus WT).

In *SREB*∆, DE genes involved with carbohydrate metabolism, including hydrolysis of O-glycosyl linkages, were enriched at 22°C (**Table 2**). Approximately 73% (33 / 45) of DE carbohydrate metabolic genes had hydrolase activity and nearly 65% (29 / 45) of these were involved with hydrolysis of O-glycosyl compounds with 72.4% (21 / 29) predicted to hydrolyze O-glycosyl linkages in chitin or glucan (**Table 2**and **S4 Table**). In *SREB*∆, the majority of DE chitinases (70%; 7/10 genes) demonstrated increased expression, whereas most DE glucanases (64%; 7/11 genes) were down-regulated (**S1 Table** and **S2 Table**). These genes were predicted to be involved with cell wall remodeling or maintenance. Deletion of *SREB* did not alter the transcription of genes involved with the biosynthesis of beta-(1,3)-glucan, beta-(1,6)-glucan, and alpha-(1,3)-glucan including *FKS1* (BDBG_00352), *RHO1* (BDBG_02597), *KRE6* (BDBG_05692), *AGS1* (BDBG_00595), and *AMY1* (BDBG_07493). Similarly, deletion of *SREB* had limited impact on chitin biosynthesis, affecting 3 of 8 chitin synthases (1.03 log_2_ decrease at 37°C for BDBG_03232, a homolog of *A*. *fumigatus csmB*; 0.84 log_2_ decrease at 24-hrs 22°C for BDBG_00955, a homolog of *A*. *fumigatus chsD*; and 1.12 log_2_ increase at 48-hrs 22°C for BDBG_07172, a homolog of *A*. *fumigatus chsB*).

Although GO analysis illuminated some common themes, detailed functional information for many DE genes was limited (**S2 Table**). For example, the majority of short chain dehydrogenases/reductases (SDRs) had unknown function (36 / 47 for catalytic activity; 37 / 49 for metabolic process ontology; 29 / 35 for oxidation-reduction process; 31 / 36 for oxidoreductase activity) (**S2 Table**). Similarly, functions for most medium chain dehydrogenases (MDRs), lyases, hydrolases, acyl carrier proteins, and acetyltransferases were unknown (**S2 Table**). Notable exceptions included an acyl carrier protein (BDBG_00048) and a GNAT acetyltransferase (BDBG_00051) involved with siderophore biosynthesis, and isocitrate lyase (BDBG_04918). *B*. *dermatitidis* encodes 2 isoforms of isocitrate lyase (non-mitochondrial—BDBG_04918, mitochondrial—BDBG_05333) and 1 malate synthase (BDBG_05338). At 24- and 48-hrs 22°C, non-mitrochondrial isocitrate lyase (BDBG_04918) and malate synthase (BDBG_05338) had > 1 log_2_ reduction in transcript compared to WT.

### Weighted gene co-expression network analysis (WGCNA)

**Fig 2:**
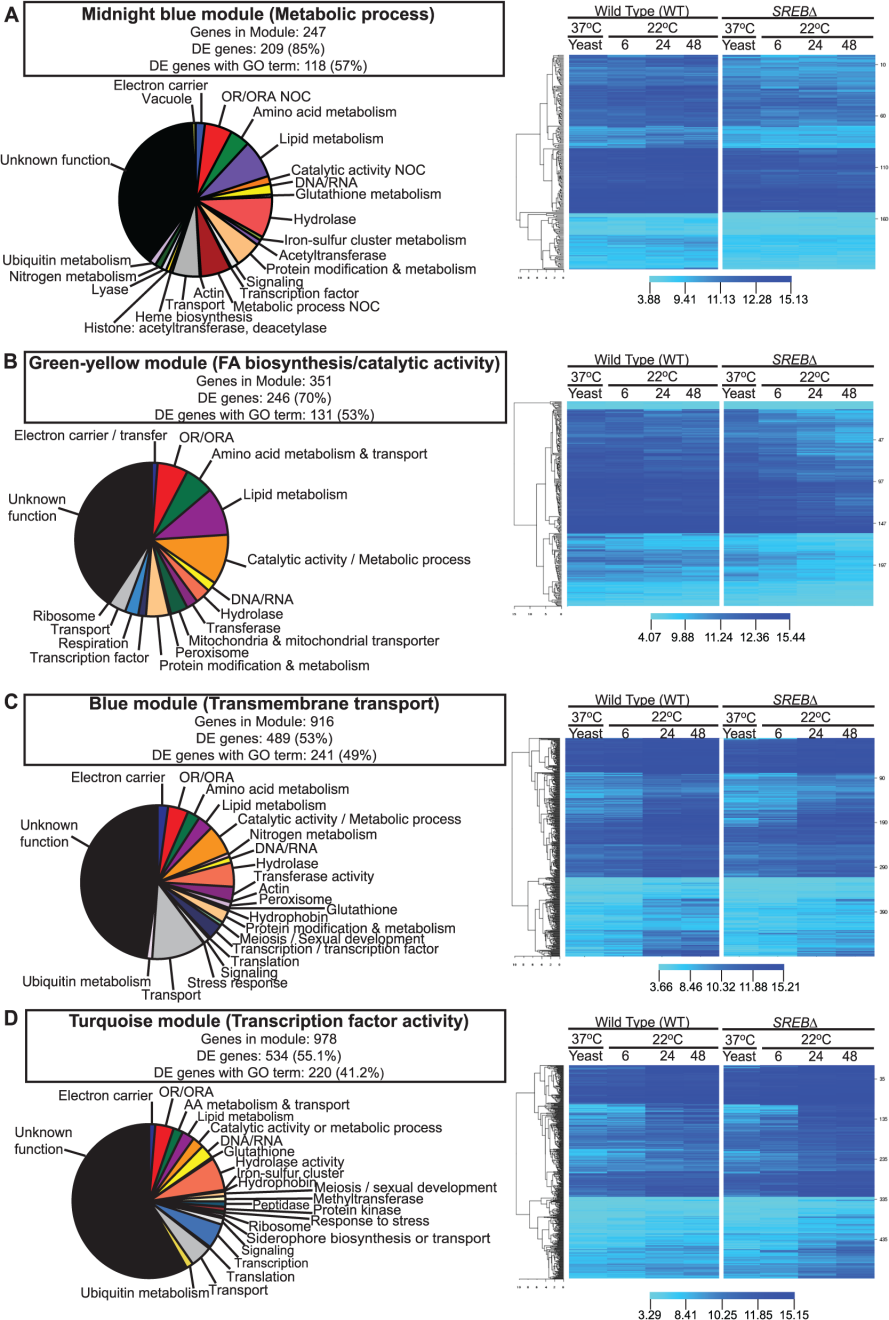
Weighted gene co-expression network analysis (WGCNA). WGCNA identified 30 color-coded modules including 4 modules in which the majority of genes were differentially expressed and enriched for GO terms. **(A)** Midnight blue, **(B)** green-yellow, **(C)** blue, and **(D)** turquoise modules were enriched metabolic process, fatty acid biosynthesis / catalytic activity, transmembrane transport, and transcription factor activity GO terms, respectively. Putative gene function was assigned by integrating GO term, tBLASTn, and pFAM analyses. Fluorescent intensity values from microarray analysis for heat maps were clustered using Euclidean distance with average linkage. Heat map analysis demonstrated distinct transcriptional patterns between *SREB*∆ and WT for each module across the time course: **(A)** midnight blue–failure of transcript for genes in *SREB*∆ to increase; **(B)** green yellow–decrease in transcript for genes in *SREB*∆; **(C)** blue–increase in transcription was attenuated for genes in *SREB*∆; and **(D)** turquoise–increased transcription for genes in *SREB*∆. Genes for the heat maps are in Supplemental Table 5.

WGCNA was performed to identify groups of co-expressed genes. All genes across all time points were included in the analysis. We identified 30 color-coded modules including 11 modules that were enriched for GO terms (**Table 3**). In 4 of these 11 modules, the majority of genes were differentially expressed (53.5%– 85.0%; **Table 3**). Moreover, 3 modules were enriched for GO terms previously identified including metabolic process (midnight blue module), fatty acid biosynthesis / catalytic activity (green-yellow module), and transmembrane transport (blue module) (**Tables 2 and 3**and **Fig 2A–2C**). Although these modules were enriched for specific GO terms, the predicted functions for DE genes within each module was diverse. Moreover, a substantial number of DE genes in each module had unknown function (**Fig 2A–2D)**. In the midnight blue module, transcription of DE genes in *SREB*∆ was lower at baseline (37°C) and failed to properly increase across the 22°C time points when compared to WT (**Fig 2A** and **S5 Table**). In contrast, DE genes in *SREB*∆ exhibited a more precipitous decrease in transcription across the time course than WT in the green-yellow module (**Fig 2B** and **S5 Table**). For the blue module, the increase in transcription across the time points at 22°C was blunted for DE genes in *SREB*∆ versus WT (**Fig 2C** and **S5 Table**). Transcription of DE genes in *SREB*∆ in the turquoise module increased during the time course compared to WT (**Fig 2D** and **S5 Table)**, which suggested these genes were derepressed. Collectively, this analysis indicated that deletion of *SREB* resulted in specific transcriptional changes at 37°C and 22°C.

**Table 3:** Weighted gene co-expression network analysis.

Module Color	Enriched GO term	*P* value	*q* value	% DE
Midnight blue	Metabolic process	3.45 x10^-10^	< 0.001	84.6% (209/247)
Green yellow	FA bio / Catalytic activity	9.48 x 10^−8^ / 6.07 x 10^−7^	< 0.001 / < 0.001	70.1% (246/351)
Turquoise	Transcription factor activity	2.94e^-6^	0.001	55.1% (534/978)
Blue	Transmembrane transport	2.69 x 10^−6^	0.001	53.5% (490/916)
Black	SC Ribo / Translation	7.15 x 10^−59^ / 4.47 x 10^−45^	< 0.001 / < 0.001	47.4% (203/428)
Light cyan	Cellular metabolic process	2.1 x 10^−11^	0.001	42.7% (102/239)
Yellow	Nucleic acid binding	2.52 x 10^−11^	< 0.001	26.6% (166/624)
Tan	Hydrolase (acid anhydrides)	0.000282	0.001	23.1% (68/295)
Pink	Signal transduction	3.07 x 10^−6^	0.001	13.9% (59/424)
Light yellow	DNA replication	5.6 x 10^−17^	< 0.001	8.7% (17/195)
Orange	Nucleosome	0.00151	0.025	0.9% (1/117)

SC Ribo is structural constituent of ribosome; FA bio is fatty acid biosynthesis. Underlined modules and GO terms were also identified in the GO enrichment analysis in Table 2.

The paucity of DE genes in modules enriched for nucleic acid binding, acid anhydride hydrolases, signal transduction, DNA replication, and nucleosome suggested that the deletion of *SREB* has limited affect on genes involved with these processes (**Table 3**).

### *SREB* is a major regulator of genes involved with iron assimilation by siderophores, but not ferric reduction

**Fig 3:**
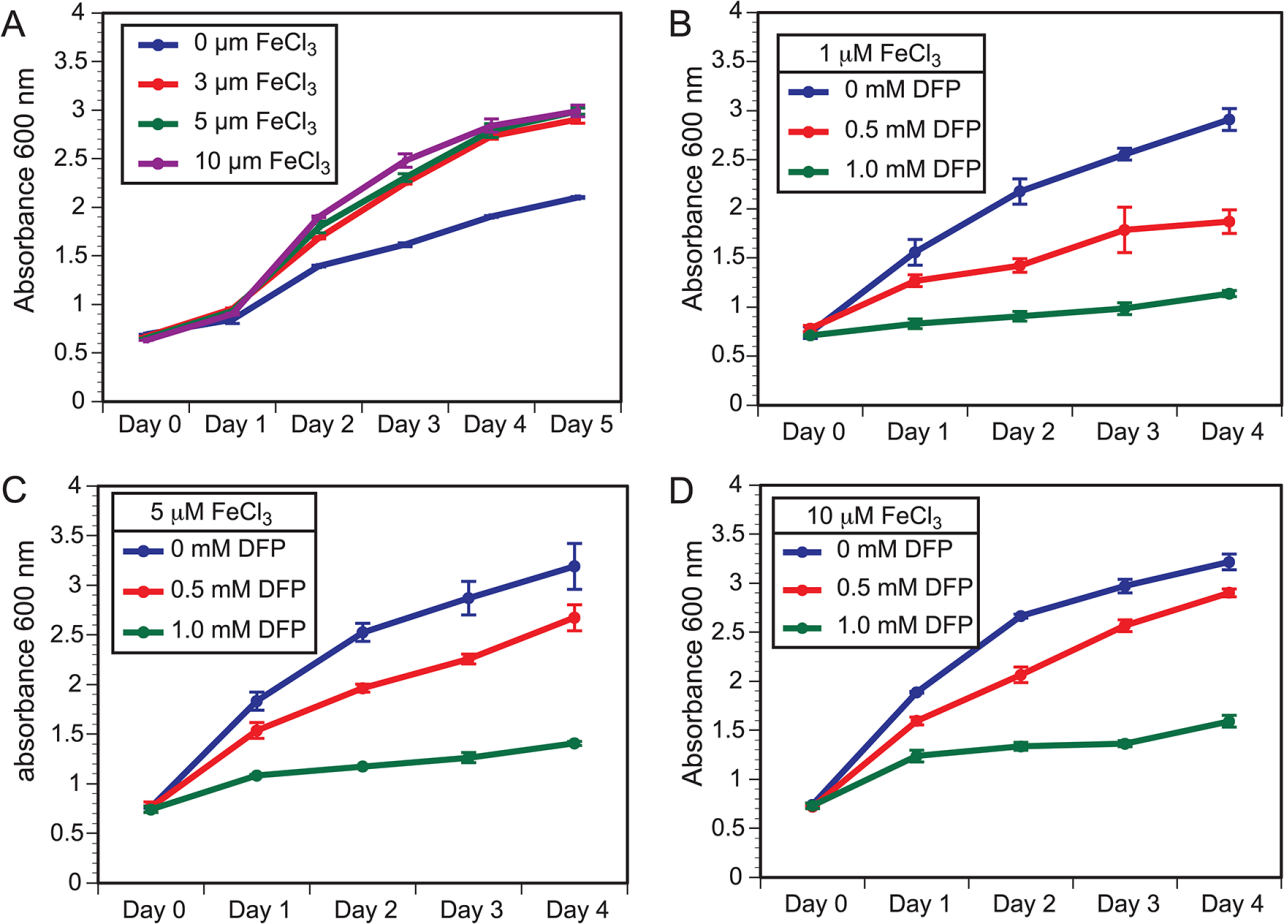
Growth of *B*. *dermatitidis* yeast is influenced by exogenous iron. **(A)** *B*. *dermatitidis* yeast were grown in iron deplete and replete liquid HMM at 37°C. **(B–D)** *B*. *dermatitidis* yeast were grown in media supplemented with 1, 5, and 10 μM FeCl_3_ along with 0–1.0 mM deferiprone (DFP), an iron chelator.

The acquisition of iron from the host or the environment is important for growth, and fungi have developed several mechanisms for iron uptake [33]. The influence of iron on the growth of *B*. *dermatitidis* is unclear and one study concluded that iron failed to enhance growth of yeast during iron starvation; however, a high concentration of ferric iron was used [34]. To further characterize the effect of iron on *B*. *dermatitidis*, yeast were grown under iron-poor media or media containing 3, 5, and 10 μM FeCl_3_. *B*. *dermatitidis* yeast exhibited slow growth under iron-deplete conditions, whereas the addition of 3 μM FeCl_3_ improved growth (**Fig 3A)**. Treatment of yeast with deferiprone (DFP), an iron chelator, impaired growth in a dose-dependent manner and the degree of growth inhibition was influenced by the concentration of exogenous iron (**Fig 3B–3D**). Collectively, these findings indicate that the acquisition of iron is important for the growth of *B*. *dermatitidis*.

**Fig 4:**
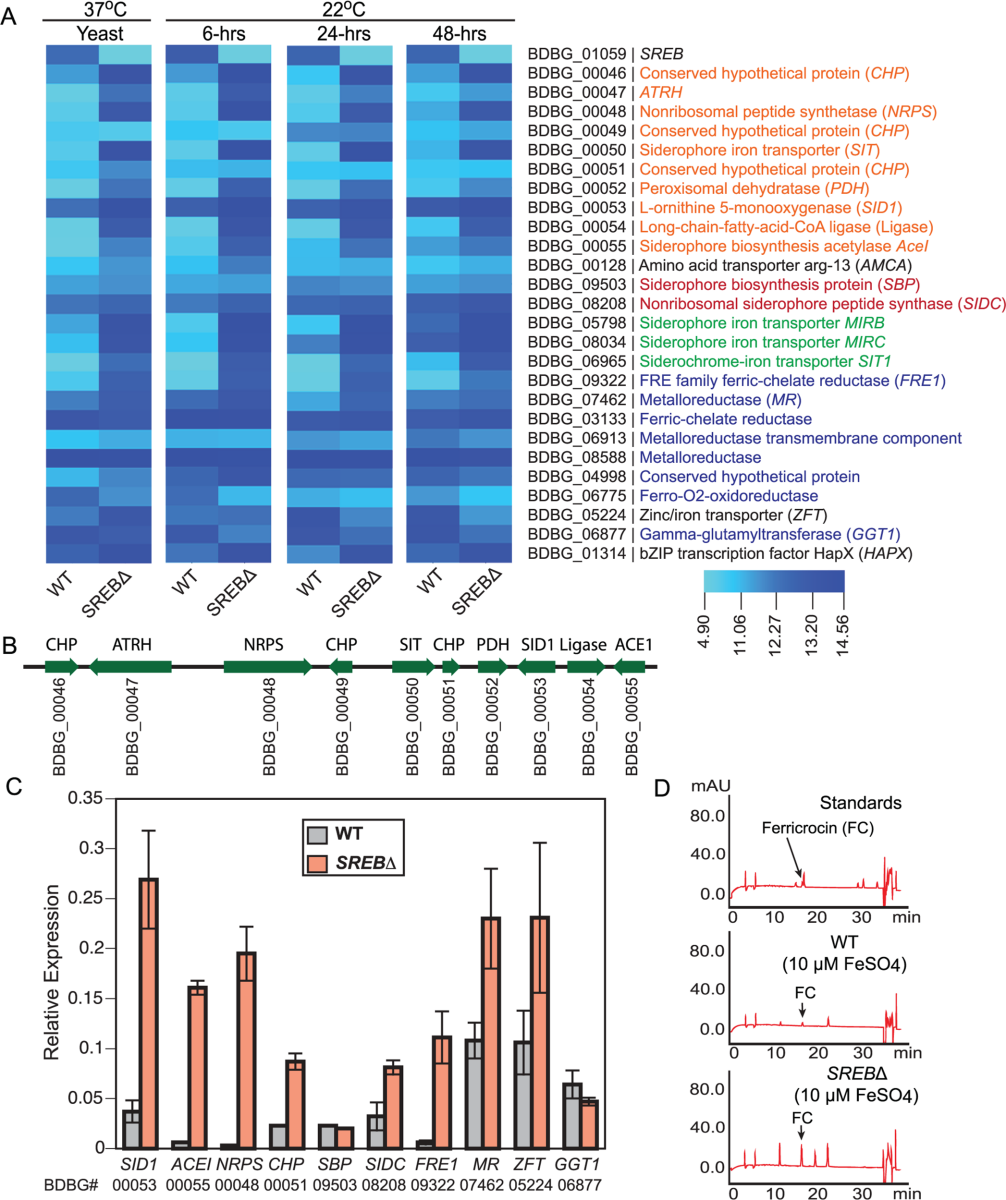
Genes in the *SREB* regulon involved with iron acquisition and homeostasis. **(A)** Heat map of genes in the *SREB* regulon involved with siderophore biosynthesis (orange text for the siderophore biosynthetic gene cluster and red text for ferricrocin biosynthetic genes), siderophore transport (green text), and ferric reduction (blue text). **(B)** Schematic of putative siderophore biosynthetic gene cluster. Locus number (BDBG #) for genes in the *Blastomyces* genome database at the Broad Institute (www.broadinstitute.org) is listed below the coding region of each gene. **(C)** Quantitative real-time PCR analysis of yeast (37°C) for a subset of genes in the *SREB* regulon involved with iron acquisition and homeostasis. The results were averaged from 2 experiments. **(D)** Reverse-phase high-pressure liquid chromatography analysis for ferricrocin, an intracellular storage siderophore, in wild-type and *SREB*∆ yeast.

Gene expression microarray analysis indicated *SREB* functioned as a major regulator of iron assimilation. *SREB*∆ failed to repress genes involved with siderophore biosynthesis and transport under iron-replete conditions at 37°C and 22°C (**Fig 4A–4C**). Moreover, a siderophore biosynthetic gene cluster (SBGC) containing 10 genes across 31.295 kb was identified (**Fig 4B**). With the exception of a gene predicted to encode a conserved hypothetical protein with unknown function (BDBG_00049), all genes in the predicted SBGC were differentially expressed in *SREB*∆ when compared to a WT isolate (**Fig 4A–4C**). DELTA-BLAST analysis against the NCBI (National Center for Biotechnology Information) database predicted that genes in the SBGC were involved with the biosynthesis of siderophores (dimerum acid and coprogen) secreted into the extracellular environment. Quantitative real-time PCR confirmed derepression of DE genes in the SBGC under iron-replete conditions in *SREB*∆ (**Fig 4C**). Two genes located outside the SBGC, BDBG_09503, BDBG_08208, were predicted to biosynthesize ferricrocin, an intracellular storage siderophore. Transcript abundance for BDBG_09503, which encodes a siderophore biosynthetic protein, was similar in *SREB*∆ and WT (**Fig 4A and 4C**). In contrast, transcript for a non-ribosomal siderophore biosynthesis peptide synthase *SIDC* (BDBG_08208) was 1.4 and 2.5-fold higher in *SREB*∆ than WT by microarray and qRT-PCR analyses, respectively (**Fig 4A and 4C**). Reverse-phase HPLC demonstrated elevated concentration of intracellular ferricrocin in *SREB*∆ compared to WT under iron-replete conditions (**Fig 4D**).

Four genes in the *B*. *dermatitidis* genome were predicted to be involved with siderophore uptake (BDBG_00050, BDBG_05798, BDBG_08034, BDBG_06965) and were derepressed in *SREB*∆ under iron-replete conditions when compared to WT (**Fig 4A–4C**). One of the siderophore transporters (BDBG_00050) was located in the SBGC (**Fig 4B**).

The reduction of ferric to ferrous iron represents an important mechanism of iron assimilation for several ascomycete fungi including *Saccharomyces cerevisiae*, *Cryptococcus neoformans*, *Candida albicans*, and *A*. *fumigatus* [35–38]. The *B*. *dermatitidis* genome encodes 6 putative ferric reductases and a glutathione-dependent ferric reductase (*GGT1*) (**Fig 4A and 4C)**. Ferric reductases are often involved with reductive iron assimilation (RIA) and function as part of a complex that includes a ferrous transporter/permease and a multicopper oxidase [39]. Deletion of *SREB* resulted in derepression of 2 of the 6 putative ferric reductases (BDBG_09322, BDBG_07462) under iron-replete conditions (**Fig 4A and 4C**). To assess the effect of *SREB* on cell surface ferric reductase activity, *SREB*∆ and WT yeast were grown in the presence of triphenyltetrazolium chloride (TTC). No enhancement of red color was observed for *SREB*∆ compared to WT under iron-replete conditions (**S4 Fig**). Translated BLAST analysis against the *B*. *dermatitidis* genome failed to yield homologs of *S*. *cerevisiae FTR1* and *A*. *fumigatus FTRA*, which encode ferrous permeases involved with RIA. Similar analysis for *Coccidioides immitis*, *Coccidioides posadasii*, and *Paracoccidioides brasiliensis* also failed to identify *FTR1*/*FTRA* homologs. *H*. *capsulatum* strains G186AR, H88, and H143 contained *FTR1*/*FTRA* homologs, whereas NAM1 and G217B did not. Microarray analysis identified a putative *B*. *dermatitidis* Zn^2+^-Fe^2+^ transporter (BDBG_05224) that was DE in *SREB*∆ (derepressed at 37°C, repressed at 22°C) (**Fig 4A and 4C**). The transcription of *B*. *dermatitidis* ferro-O_2_-oxidoreductase (BDBG_06775), which is a homolog of *S*. *cerevisiae* FET5 multicopper oxidase, was repressed in *SREB*∆ (versus WT) at 37°C and 22°C under iron-replete conditions (**Fig 4A**). Collectively, these data indicate that *SREB* has a limited impact on the transcription of genes involved with ferric reduction.

Similar to *H*. *capsulatum*, the genome of *B*. *dermatitidis* encodes a γ-glutamyltransferase (*GGT1*; BDBG_06877), a glutathione-dependent ferric reductase, whose mechanism for iron acquisition is independent of RIA [40]. *B*. *dermatitidis GGT1* transcript abundance in *SREB*∆ is similar to WT at 37°C, but is decreased at 22°C under iron-replete conditions (**Fig 4A and 4C**). These data suggest that unlike siderophore biosynthesis and transport, *SREB* does not repress *GGT1* transcription under iron-replete conditions.

### Deletion of *SREB* alters lipid metabolism

**Fig 5:**
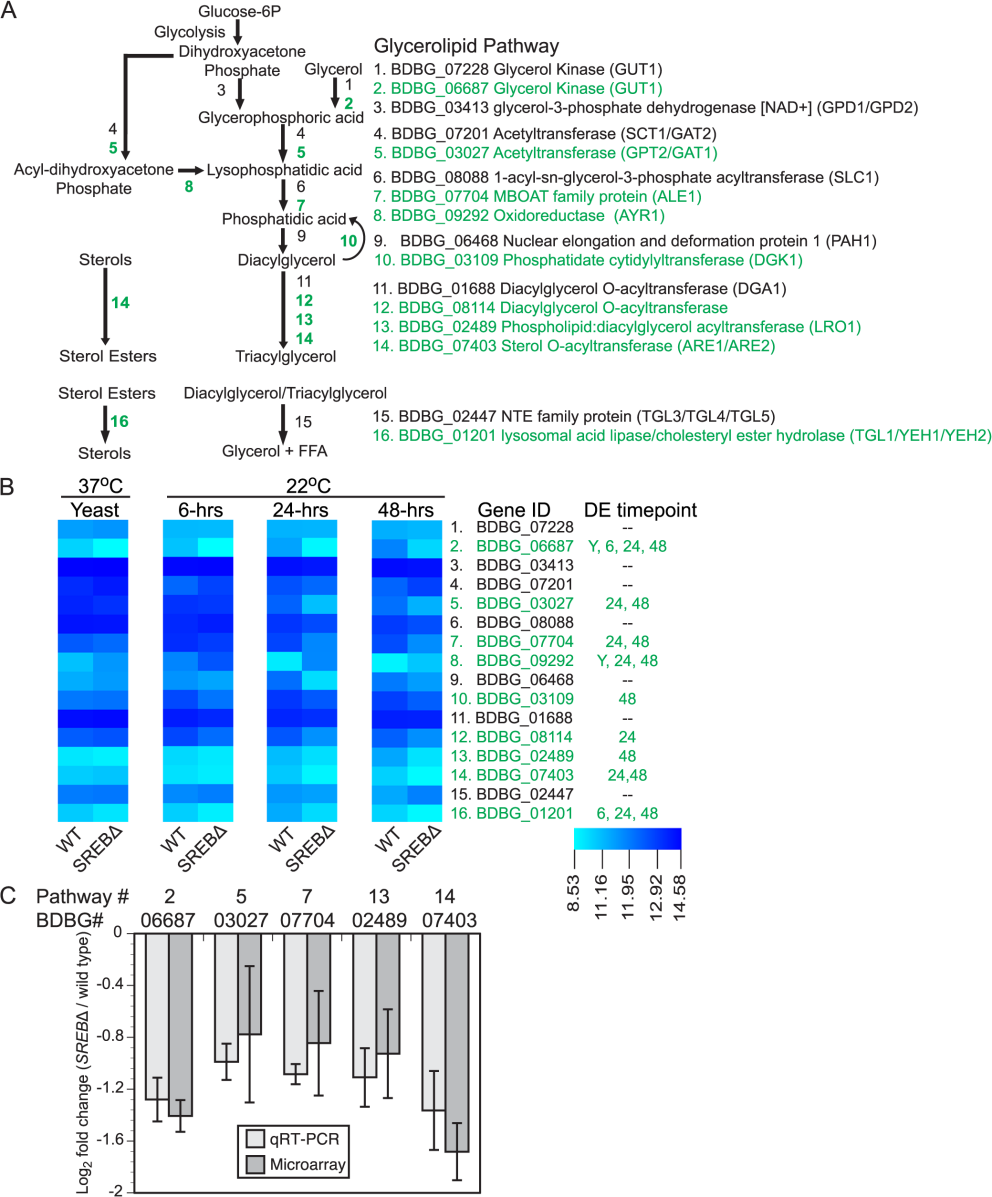
Deletion of *SREB* affects the transcription of genes in the glycerolipid biosynthetic pathway. **(A)** Schematic of the glycerolipid biosynthetic pathway in *B*. *dermatitidis*. Putative lipases and esterases involved with sterol, diacylglycerol, and triacylglycerol breakdown are also shown. Differentially expressed (DE) genes are labeled in green. **(B)** Heat map of fluorescent intensity values (log_2_) for genes in glycerolipid biosynthetic pathway in wild-type (WT) and *SREB*∆ isolates. Genes are arranged in the same order as in (A). The time point at which gene transcription was DE in *SREB*∆ versus WT is adjacent to the heat map. DE genes are labeled in green. **(C)** Quantitative RT-PCR analysis of a subset of DE glycerolipid biosynthetic genes at 48-hrs 22°C. Log2 fold change values (*SREB*∆ versus wild-type) for qRT-PCR were compared to gene expression microarray data for BDBG_06687 (glycerol kinase), BDBG_03027 (acetyltransferase), BDBG_07704 (MBOAT family protein), BDBG_02489 (Phospholipid:diacylglycerol acetyltansferase), BDBG_07403 (Sterol-O-acyltransferase). The qRT-PCR results were averaged from 2 experiments.

**Fig 6:**
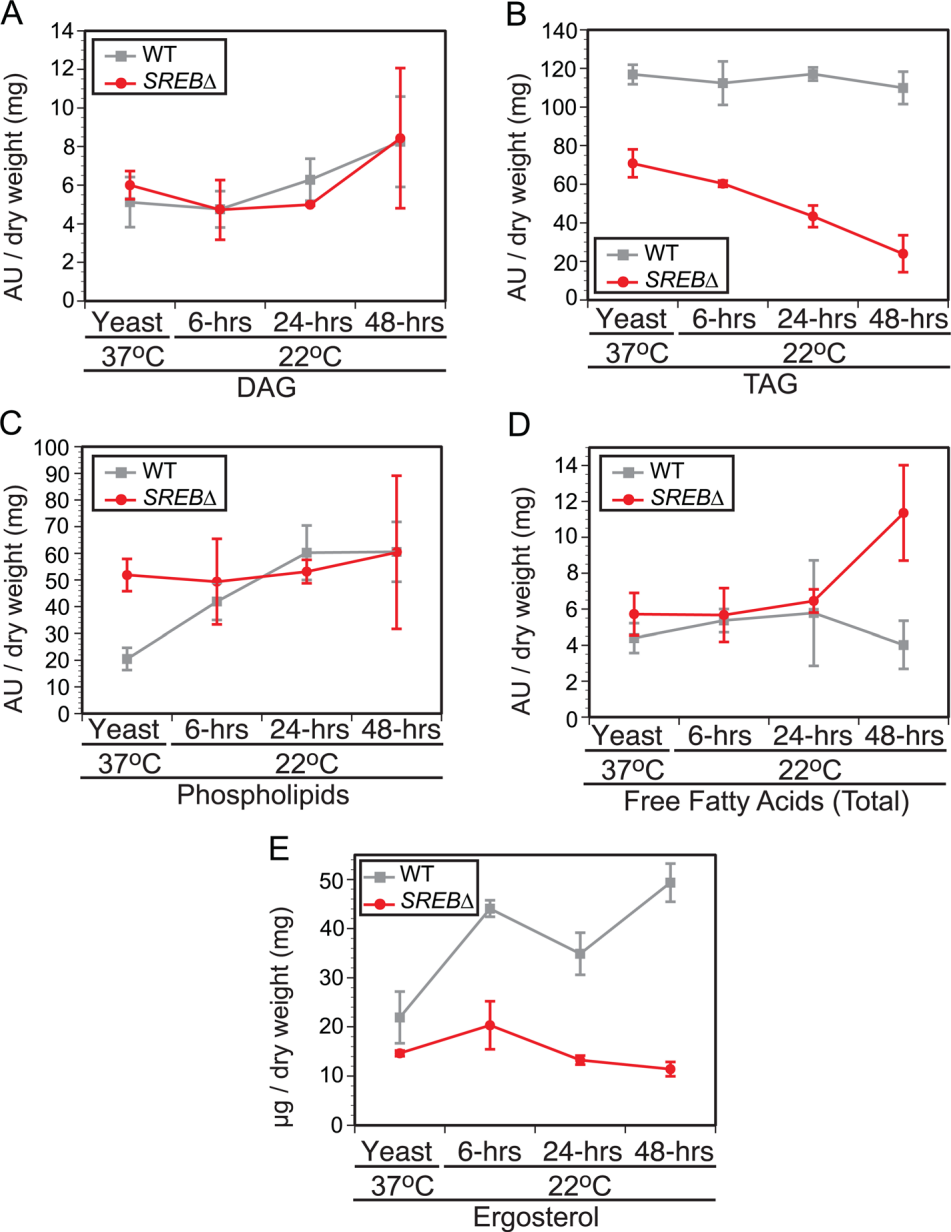
Impact of *SREB* on lipid biosynthesis. Gas chromatographic analysis of **(A)** diacylglycerol (DAG), **(B)** triacylglycerol (TAG), **(C)** phospholipids, **(D)** free fatty acids, and **(E)** ergosterol concentrations in wild-type (WT) and *SREB*∆ isolates. The results were averaged from 2 biological replicates for WT and *SREB*∆. Each biological replicate consisted of 2 technical replicates. The fatty acid abundance reported reflects the fatty acids present at end of protocol. Peak areas, which are denoted as arbitrary units (AU) were normalized by dry weight in milligrams.

GO enrichment and WGCNA analyses suggested that deletion of *SREB* affected lipid metabolism at 37°C and 22°C. To investigate for defects in lipid biosynthesis, glycerolipids (triacylglycerol (TAG) and diacylglycerol (DAG)), phospholipids, free fatty acids, and ergosterol were extracted from WT and *SREB*∆ at 37°C and 6, 24, and 48-hrs at 22°C. Gene expression microarray analysis indicated 8 of 14 genes in the glycerolipid pathway involved with TAG biosynthesis were DE in *SREB*∆ versus WT (**Fig 5A and 5B**). Seven DE genes had decreased transcript abundance at 22°C, whereas a homolog of *S*. *cerevisiae AYR1* (BDBG_09292) had increased transcription (**Fig 5A and 5B**). Quantitative RT-PCR analysis validated a subset of these DE genes; log_2_ fold changes were similar for qRT-PCR and microarray analyses at 48-hrs 22°C (**Fig 5C**). Gas chromatographic analysis of extracted lipids from *SREB*∆ suggested decreased TAG concentrations at 37°C and 22°C, and similar levels of DAG compared to WT (**Fig 6A and 6B**).

Deletion of *SREB* altered the transcription for 14 of 21 genes predicted to be involved with the biosynthesis of ergosterol (**S5 Fig**), a medically important sterol in the plasma membrane targeted by polyene and azole antifungals. Nearly all DE genes (12 / 14) in the ergosterol biosynthetic pathway had decreased transcription. In *SREB*∆, ergosterol concentrations were decreased at 37°C and 22°C compared to the WT isolate (**Fig 6E**). Moreover, ergosterol concentrations failed to increase in *SREB*∆ following a drop in temperature to 22°C (**Fig 6E**). To assess the effect of decreased ergosterol concentrations in *SREB*∆ to antifungal drugs, disc diffusion testing using amphotericin B deoxycholate (0, 0.5, 1, 2.5, 5, 10 μg) and voriconazole (0, 0.06, 0.125, 0.25, 0.5, 1 μg) was performed. No difference in antifungal drug susceptibility was observed in *SREB*∆ versus WT at 37°C (**S6 Fig**). Antifungal susceptibility testing at 22°C was precluded because *SREB*∆ exhibited a growth defect at this temperature; it failed to expand by radial growth or accumulate significant biomass [18].

In contrast to TAG and ergosterol, the concentration of phospholipids in *SREB*∆ and WT were similar following a drop in temperature from 37°C to 22°C (**Fig 6C**). Alterations in free fatty acid content were restricted to a single time point (48-hrs 22°C) in *SREB*∆ (**Fig 6D**), which was due to an increase in stearic acid (18:0) without alteration in oleic acid (18:1n9).

**Fig 7:**
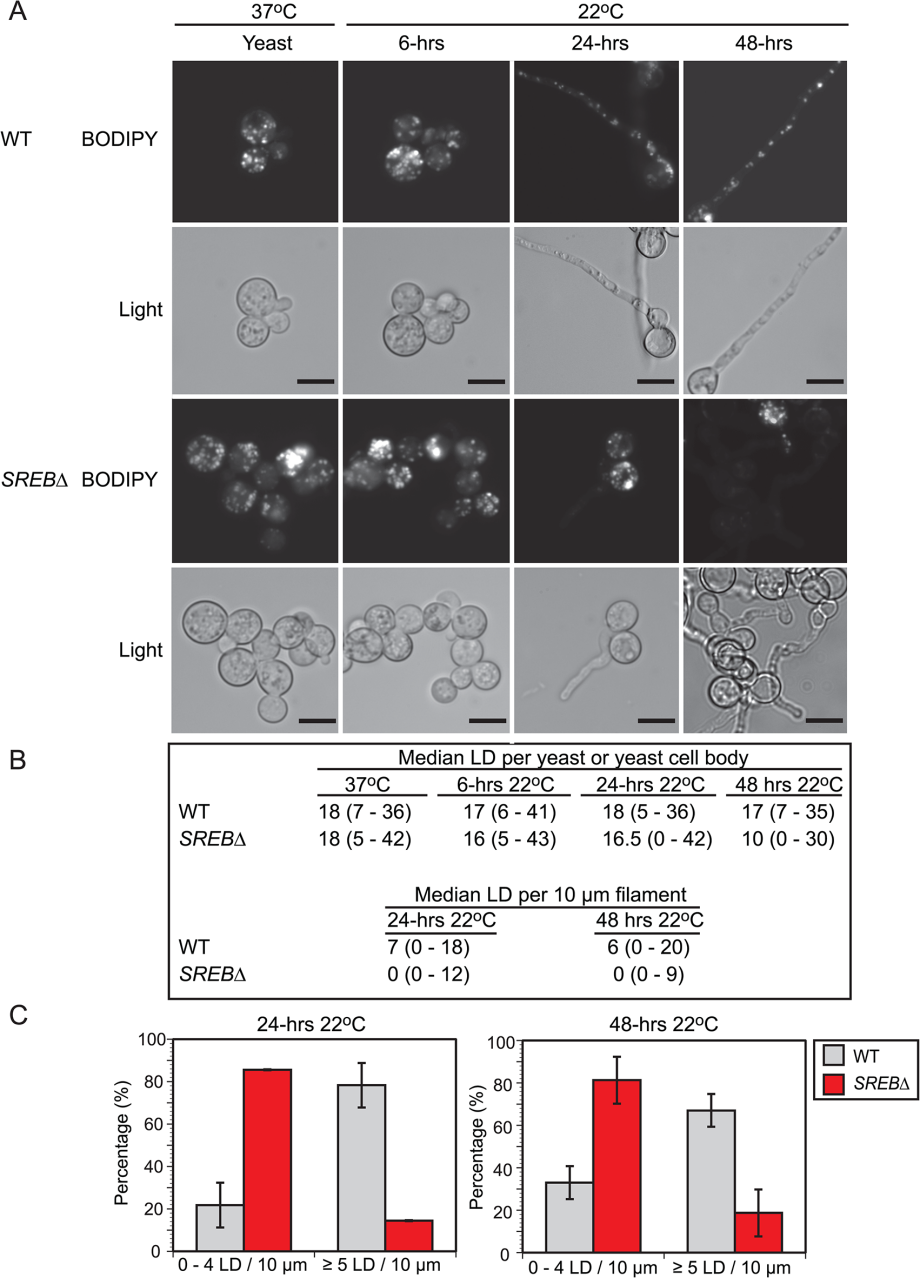
Lipid droplets in wild type and *SREB*∆. (**A**) BODIPY 493/503 was used to stain for lipid droplets (LD) in wild type (WT) and *SREB*∆ at 37°C and 6, 24, and 48-hrs following a drop in temperature to 22°C. Corresponding bright field microscopic images (light) are below the fluorescent images. Scale bar is 10 μm (**B**) Median number of lipid droplets per yeast, yeast cell body, and 10 μm filament were quantified at 37°C and 22°C (range is in parentheses). To adjust for differences in filamentous growth at 24 and 48-hrs 22°C, LDs were quantified per 10 μm segment along the length of the filament. LDs were quantified from 50 cells in duplicate. (**C**) Percentage of filaments that contained 0–4 or ≥ 5 LDs per 10 μm segment at 24 and 48-hrs 22°C.

The reduction in TAG and ergosterol in *SREB*∆ prompted investigation for alteration in lipid droplets at 37°C and 22°C. Lipid droplets are organelles that consist of a neutral lipid core of TAG and sterol esters surrounded by a phospholipid monolayer intercalated with proteins [41]. Sterol esters are derived from ergosterol and intermediates in the ergosterol biosynthetic pathway [42]. *SREB*∆ was analyzed for defective lipid droplet formation using BODIPY, a fluorescent dye that is specific for lipid droplets (LDs) [43]. LDs were quantified in cells with yeast morphology at 37°C and 6-hrs 22°C. At 24 and 48-hrs 22°C, LDs in the yeast cell body as well as the emerging filament (germ tube or hyphae) were quantified. To adjust for the differences in filament length for *SREB*∆ compared to WT, LDs were quantified per 10 μm segments along the length of the filament. At 37°C and 6-hrs 22°C, there were no differences in LD abundance in yeast cells between *SREB*∆ and WT (**Fig 7A and 7B**). At 24-hrs 22°C, the median LD abundance was sharply reduced in *SREB*∆ filaments versus WT (0 versus 7); however, the median number of LD per yeast cell body for *SREB*∆ was similar to WT (16.5 versus 18) (**Fig 7A and 7B**). At 48-hrs 22°C, the median number of LDs per 10 μm segment (0 versus 6) and yeast cell body (10 versus 17) was decreased in *SREB*∆ compared to WT (**Fig 7A and 7B**). At 24 and 48-hrs 22°C, more than 80% of 10 μm segments analyzed for *SREB*∆ had ≤ 4 LD, whereas for WT, less than 33% of 10 μm segments had 4 or fewer LD (**Fig 7C**).

**Fig 8:**
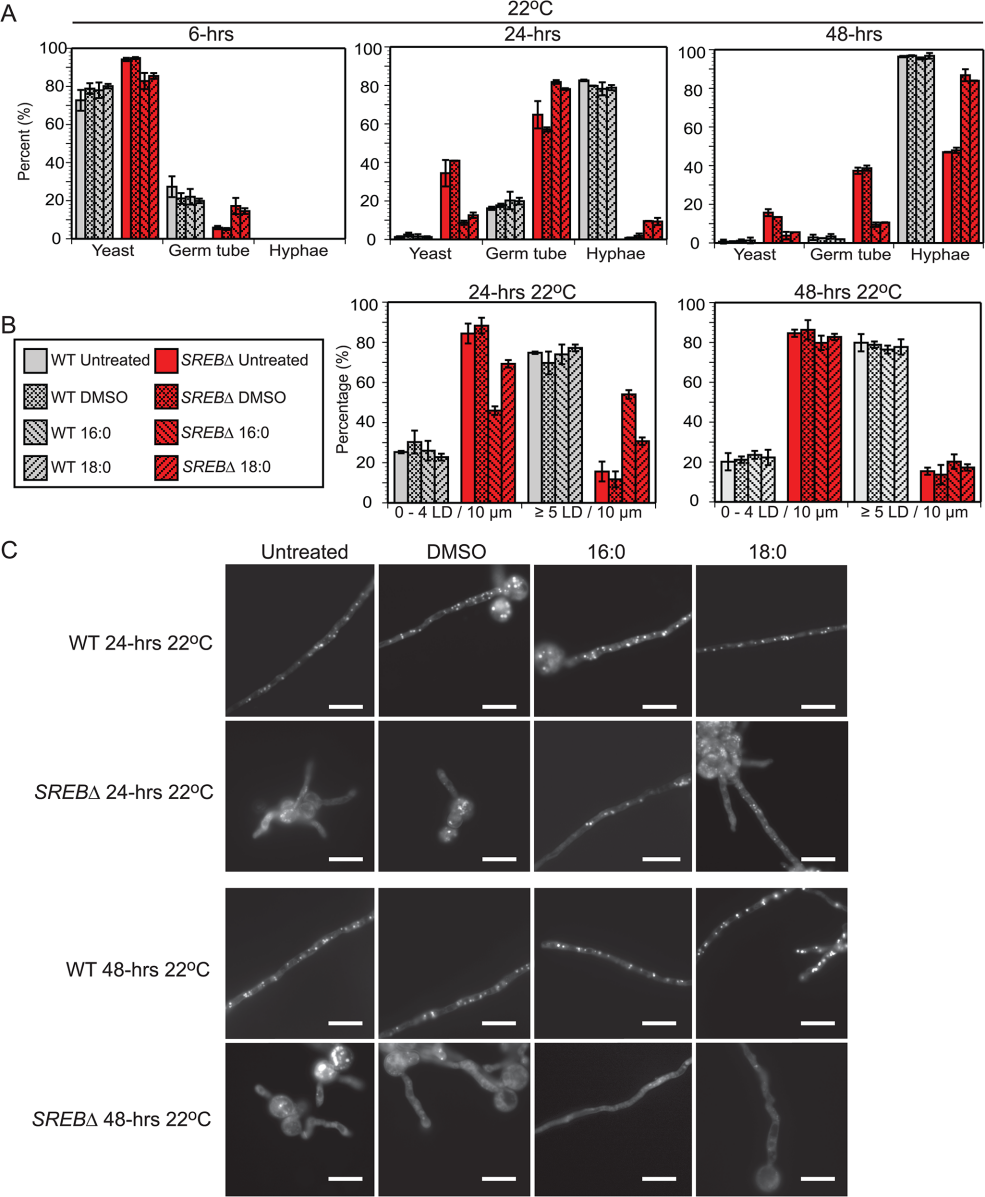
Fatty acid supplementation for wild type and *SREB*∆. (**A**) Percentage of WT and *SREB*∆ with yeast morphology, germ tube development, and hyphae at 22°C for cells grown in media supplemented with 0.5 mM palmitic acid (16:0) or 0.5 mM stearic acid (18:0). Controls included DMSO only and cells grown in media without DMSO or saturated fatty acid (untreated). At least 200 cells were counted in duplicate. Results were averaged from 2 independent experiments. **(B**) Percentage of filaments that contained 0–4 or 5 LDs per 10 μm segment at 24 and 48-hrs 22°C for WT and *SREB*∆ cells (untreated, DMSO only, 16:0 and 18:0). LDs were quantified per 10 μm segment along the length of filaments from at least 30 cells. Results were averaged from 2 independent experiments. (**C**) BODIPY 493/503 staining of lipid droplets at 24 and 48-hrs 22°C for WT and *SREB*∆ cells (untreated, DMSO only, 16:0 and 18:0). Scale bar equals 10 μm.

In eukaryotic cells, fatty acids (FAs) serve as an important substrate for TAG and sterol ester biosynthesis [44,45]. To investigate if exogenous fatty acids can influence the phase transition or LD formation, *SREB*∆ cells were treated with saturated (16:0, 18:0) and unsaturated (16:1n7, 18:1n9) fatty acids. Experiments with exogenous TAG were not performed because fungal cells are unable to uptake this glycerolipid. The poor solubility of saturated FAs in aqueous solutions necessitated dissolving 16:0 and 18:0 in DMSO prior to supplementation of iron-replete HMM. Treatment of *SREB*∆ with 0.5 mM palmitic (16:0) or 0.5 mM stearic (18:0) acid accelerated the morphologic switch at 22°C compared to control strains (*SREB*∆ untreated, *SREB*∆ DMSO) without affecting the phase transition of WT cells (untreated, DMSO, 16:0, 18:0) (**Fig 8A**). At 24-hrs 22°C, exogenous 16:0 and 18:0 increased germ tube formation in *SREB*∆ versus *SREB*∆ untreated and *SREB*∆ DMSO (**Fig 8A**). At 24 and 48-hrs 22°C, *SREB*∆ treated with 16:0 or 18:0 exhibited increased conversion to hyphae and decreased number of yeast cells compared to controls (*SREB*∆ untreated, *SREB*∆ DMSO) (**Fig 8A**). The morphology of yeast cells at 37°C was unaffected by 16:0 or 18:0 (**S7 Fig**). Although DMSO did not affect the phase transition (**Fig 8A**), higher concentrations of DMSO needed to solubilize ≥ 1 mM 16:0 or 18:0 hindered the morphologic switch to mold for WT and *SREB*∆. BODIPY staining demonstrated that exogenous 16:0 and 18:0 restored LDs in the growing filaments of *SREB*∆ at 24-hrs 22°C (**Fig 8B and 8C**). Median LD per 10 μm increased from 1 for *SREB*∆ controls (untreated, DMSO) to 5 for *SREB*∆ 16:0 and 2.5 for *SREB*∆ 18:0. Moreover, the percentage of 10 μm filament segments with 5 LDs increased 4-fold for *SREB*∆ 16:0 and 2.2-fold for *SREB*∆ 18:0 compared to controls (*SREB*∆ untreated, *SREB*∆ DMSO) (**Fig 8B**). The increase in LDs was transient and limited to the 24-hr 22°C time point (**Fig 8A–8C**). At 48-hrs 22°C, the median number of LDs per 10 μm for *SREB*∆ 16:0 and 18:0 declined to 2 and the percentage of 10 μm filament segments with ≤ 4 LDs increased (**Fig 8A–8C**). Exogenous 16:0 or 18:0 did not affect the number of lipid droplets for *SREB*∆ at 37°C and 6-hrs 22°C (**Fig 8B and 8C**, and **S7 Fig**). WT cells treated with 16:0 or 18:0 had similar number of LDs as controls (median 6–7 LD per 10 μm filament segment at 24 and 48-hrs 22°C) (**Fig 8B and 8C**). *SREB*∆ cells treated with 0.5 mM oleic acid (18:1n9) exhibited similar morphologic and LD defects as untreated *SREB*∆ cells at 48-hrs 22°C (**S8 Fig**). Treatment of cells with 0.5 mM palmitoleic acid (16:1n7) was lethal for WT and *SREB*∆; reducing 16:1n7 concentrations to 0.250 mM or 0.125 mM did not improve cell viability (**S8 Fig**).

### SREB directly binds and regulates the transcription of genes with disparate functions

ChIP-qPCR was used to identify genes bound and directly regulated by SREB *in vivo*. For ChIP, SREB was engineered to contain an in-frame 3x-hemagglutinin (HA) epitope tag at the C-terminus. To minimize mis-expression or abnormal cellular localization, *SREB-3xHA* was placed under the control of its native promoter and contained the 3’-untranslated region, respectively. Retransformation of *SREB*∆ with *SREB-3xHA* complemented the defects in phase transition (**S9 Fig**) and siderophore biosynthesis indicating the construct was functional. Western blot analysis using a ChIP-grade rabbit polyclonal HA antibody demonstrated specificity for 26199 strains transformed with *SREB-3xHA* and absence of antibody hybridization for wild type 26199 (**S9 Fig**). To identify potential sites of DNA binding by SREB, gene expression microarray data were integrated with genome-wide *in silico* analysis of extended GATA binding motifs (ATC-w-gAta-a) in promoters of differentially expressed genes; approximately 12% of DE genes possessed an extended GATA motif. Use of the classic GATA motif (A/T-GATA-A/G) was limited because this motif was found upstream of nearly all genes (DE and non-DE) in the *B*. *dermatitidis* genome [18].

**Fig 9:**
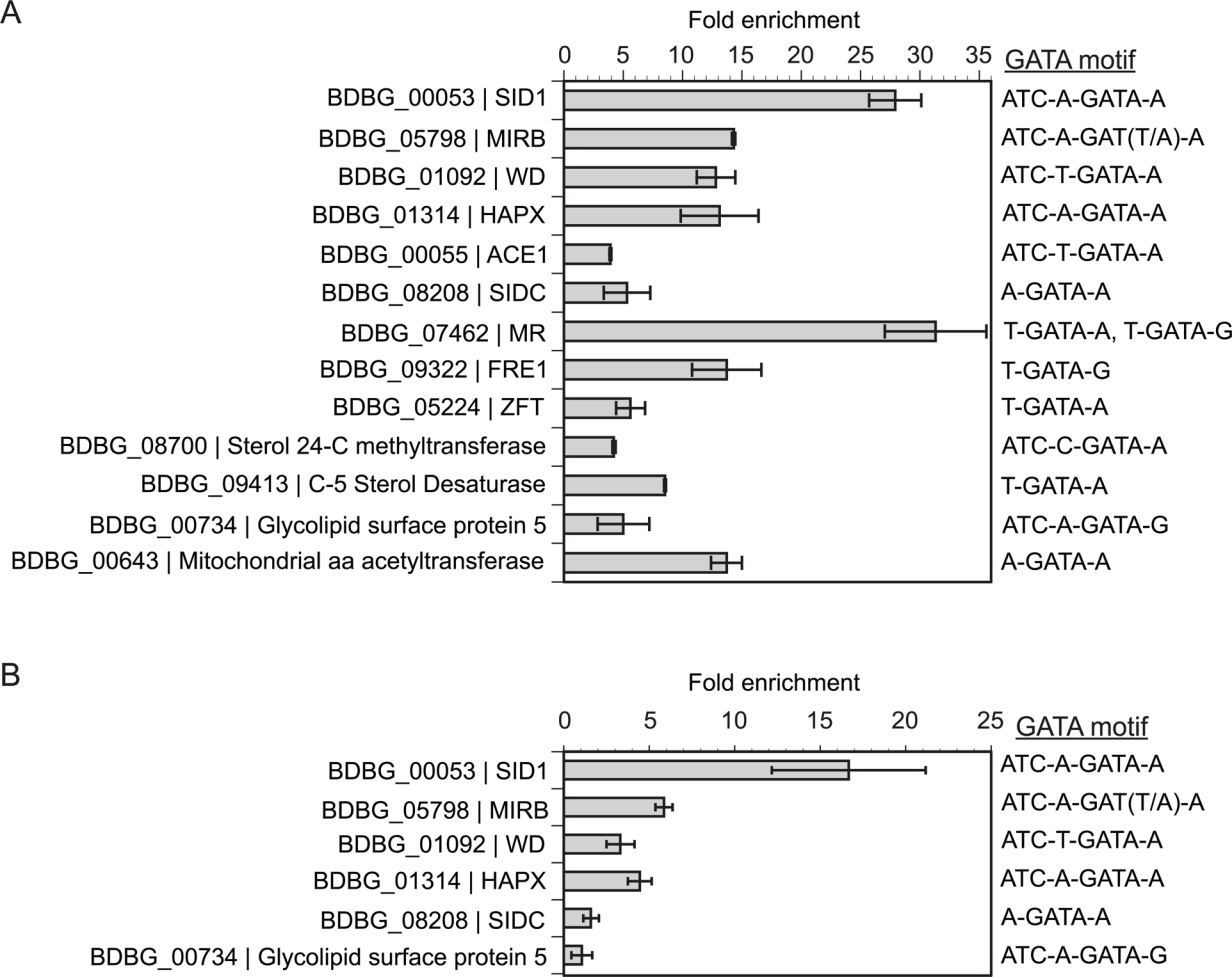
Chromatin immunoprecipitation with quantitative real-time PCR (ChIP-qPCR). **(A)** Enrichment for binding of SREB-3xHA to promoter regions containing GATA binding motifs in *B*. *dermatitidis* yeast at 37°C. **(B)** Enrichment binding of SREB-3xHA to promoter regions containing GATA binding motifs in *B*. *dermatitidis* cells at 48-hrs following a drop in temperature from 37°C to 22°C. ChIP-qPCR was performed using SREB that was engineered to contain a C-terminal 3x-hemagglutinin tag. Integration of gene expression microarray data with genome-wide GATA motif analysis was used to identify putative GATA transcription factor binding sites. All genes analyzed were differentially expressed and contained a GATA motif (ATC-w-gAta-a or A/T-GATA-A/G) in the upstream promoter. For *MIRB*, the upstream motif in strain 26199 was ATC-A-GATT-A, whereas in other strains including SLH14081, ER-3, and ATCC 18188, the motif was ATC-A-GATA-A. BDBG is the locus number for genes in the *B*. *dermatitidis* genome database at the Broad Institute (www.broadinstitute.org).

Using this integrated approach, we identified a subset of genes bound and regulated by SREB-3xHA under iron-replete conditions (10 μM FeSO_4_) using ChIP-qPCR (**Fig 9A and 9B**). These included genes involved with extracellular (*SID1*, *ACE1*) and intracellular (*SIDC*) siderophore biosynthesis, siderophore transport (*MIRB*), ferric reduction (*FRE1* and *MR*), ferrous iron/zinc transporter (*ZFT*), a WD-repeat protein of unknown function (*WD*), and a bZIP transcription factor (*HAPX*) (**Fig 9A and 9B**). For ChIP-qPCR, SREB-3xHA did not bind the *GAPDH* promoter, which lacked GATA motifs and served as a negative control. ChIP-seq analysis of yeast grown in iron-replete media (10 μM FeSO_4_) also demonstrated enrichment of SREB-3xHA binding for genes located in the siderophore biosynthetic gene cluster including BDBG_00046 (conserved hypothetical protein), BDBG_00047 (*ATRH*), BDBG_00048 (*NRPS*), BDBG_00053 (*SID1*), BDBG_00054 (long chain fatty acid CoA ligase), and BDBG_00055 (*ACE1*) (**S10 Fig** and **S6 Table**). In addition, SREB-3xHA bound to the promoters of *SIDC* (BDBG_08208) and siderophore transporters *MIRB* (BDBG_05798), *MIRC* (BDBG_08034), and *SIT1* (BDBG_06965) (**S10 Fig** and **S6 Table**). ChIP-seq also showed enrichment of SREB-3xHA upstream of genes unrelated to iron homeostasis including a glycolipid surface protein predicted to have 1,3-beta-glucanosyltransferase activity (BDBG_000734), GNAT acyltransferase (BDBG_06912), a thioesterase-domain containing protein (BDBG_07971), and a cation diffusion facilitator (BDBG_07469) (**S11 Fig** and **S6 Table**). These genes were DE in the microarray analysis (**S6 Table**). In addition, SREB-3xHA was enriched upstream of an amino acid acetyltransferase, BDBG_00643, which is derepressed in *SREB*∆ and is predicted to be involved in the biosynthesis of ornithine. ChIP-qPCR confirmed enrichment of a subset of these genes including the glycolipid surface protein (BDBG_000734) and amino acid acetyltransferase (BDBG_00643) (**Fig 9A and 9B,** and **S6 Table**).

The reduction of ergosterol and TAG concentrations in *SREB*∆ prompted investigation of SREB-3xHA binding to a subset of differentially expressed genes in the ergosterol and glycerolipid biosynthetic pathways. Of the 14 DE genes in the ergosterol pathway, only *ERG6*, which encodes a sterol 24-C methyltransferase (BDBG_08700) contained an extended GATA motif in the promoter region. This gene was DE at 37°C (0.80 log_2_ decrease) and ChIP-qPCR demonstrated 4.2-fold enrichment in yeast (**Fig 9A**). Surprisingly, enrichment for this gene was not observed by ChIP-seq analysis at 37°C. This discrepancy may be related to differences in chromatin shearing. However, SREB-3xHA was enriched by ChIP-seq analysis upstream of a C-5 sterol desaturase (BDBG_09413, -1.0 Log_2_ fold change at 37°C) in the ergosterol biosynthetic pathway (**S5 Fig**) and binding was confirmed by ChIP-qPCR (**Fig 9A** and **S6 Table**). Differentially expressed genes involved with conversion of DAG to TAG in the glycerolipid biosynthetic pathway (BDBG_02489, BDBG_07403, BDBG_08114) were analyzed by ChIP-qPCR at 48-hrs 22°C. These genes possessed classic GATA motifs but lacked extended motifs. No enrichment was observed for BDBG_02489 and BDBG_07403 at 22°C. Analysis of BDBG_08114 was precluded due to difficulty in designing high-quality primers targeting the region containing the GATA motif.

### Overexpression of HAPX alters the phase transition

**Fig 10:**
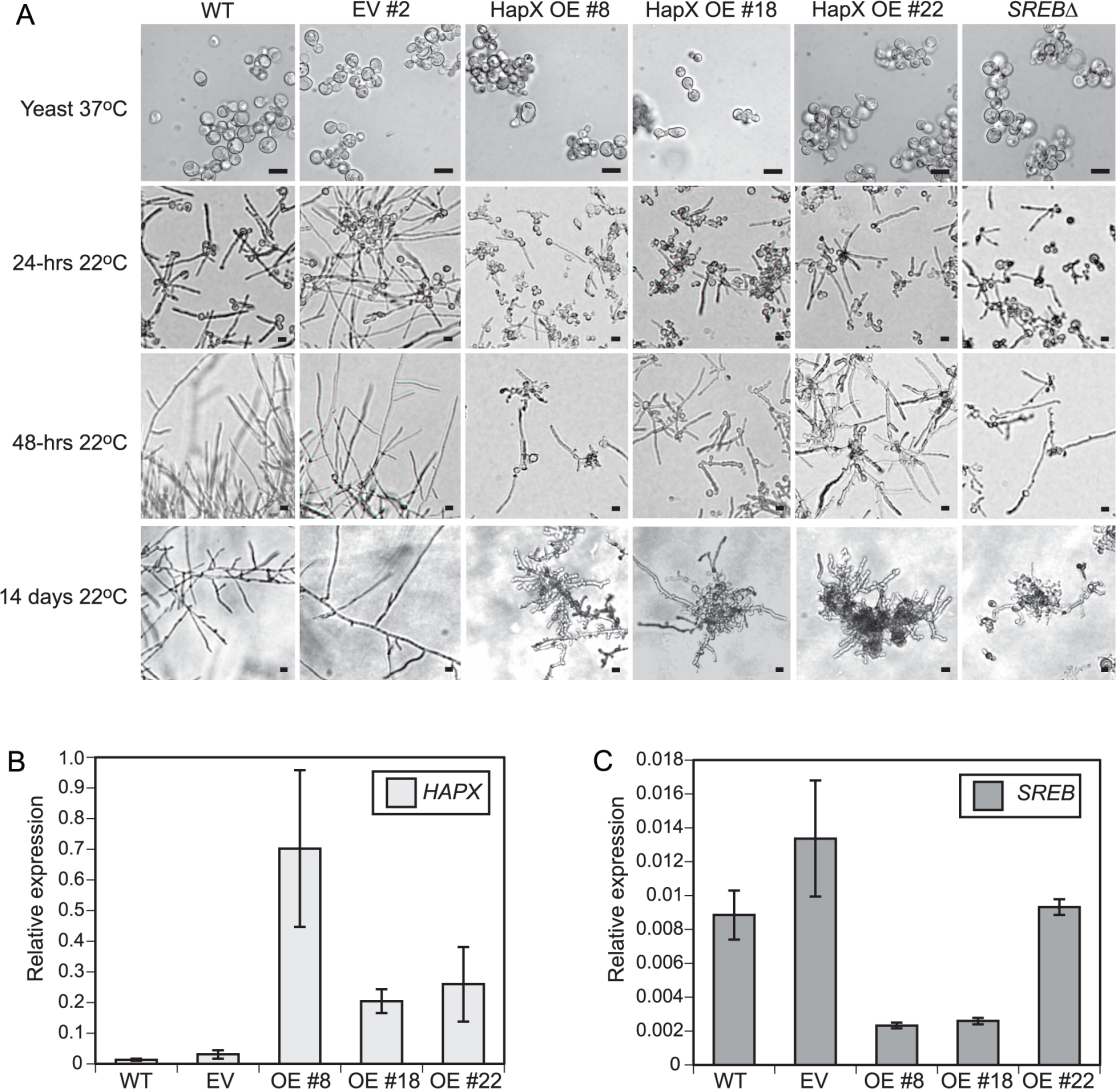
*HAPX* overexpression affects the phase transition in *B*. *dermatitidis*. **(A)** Similar to *SREB*∆, *HAPX* overexpression (OE) strains #8, #18, and #22 grew as yeast at 37°C, but failed to complete conversion to mold following a downshift in temperature to 22°C. In contrast, wild-type (WT) and empty vector (EV) strains converted to mold at 22°C. Scale bar is 10 μm. **(B)** Quantitative RT-PCR analysis of *HAPX* transcript abundance in controls (WT, EV) and overexpression (OE# 8, OE#18, OE#22) strains. *HAPX* OE strains had 15.5–53.1-fold increased *HAPX* transcript abundance compared to controls. **(C)** Quantitative RT-PCR demonstrated a 3.4–5.8-fold decrease in *SREB* transcript abundance in OE strains #8 and #18 compared to WT and EV controls. *SREB* transcript abundance in OE #22 exhibited a 0.9–1.4-fold change compared to controls (WT, EV). All strains were grown in liquid HMM supplemented with 10 μM FeSO_4_. Quantitative RT-PCR results were averaged from 2 experiments.

Gene expression microarray data indicated that *SREB* altered the phase transition independent of *VMA1* and *HGRA*; these genes are not DE in *SREB*∆. To test the importance of a subset of SREB-bound genes on the phase transition, we overexpressed *WD* and *HAPX* in wild-type strain 26199 under the control of an H2B promoter. *WD* was selected for overexpression because WD-repeat proteins can be involved with transcriptional regulation and affect fungal development [46,47]. *HAPX* is also involved with transcriptional regulation and in *Aspergillus* spp., it is in a negative regulatory circuit with *SREA*, a homolog of *SREB* [48,49]. These genes were overexpressed rather than silenced because they were derepressed in *SREB*∆. The H2B promoter was active at 37°C, 22°C, and during the phase transition when measured by qRT-PCR (Ct values 21 at 37°C and 22°C for H2B transcript). *WD* overexpression strains grew as yeast and converted normally to mold at 22°C. *HAPX* overexpression strains (OE 8, 18, 22) were morphologically similar to *SREB*∆ following a drop in temperature from 37°C to 22°C (**Fig 10A**). Quantitative RT-PCR analysis demonstrated 15.5–53.1-fold increase in *HAPX* transcript abundance in OE strains compared to WT (**Fig 10B**). *SREB* transcript abundance was decreased in OE 8 and OE 18, but was similar to WT and empty vector for OE 22 (**Fig 10C**). In *HAPX OE* 8 and 18, the reduction in *SREB* transcript was unstable and normalized to WT levels over serial passage; however, the morphologic defect and *HAPX* overexpression persisted. Unlike deletion of *SREB*, overexpression of *HAPX* did not adversely affect LD formation at 24 and 48-hrs 22°C.

**Fig 11:**
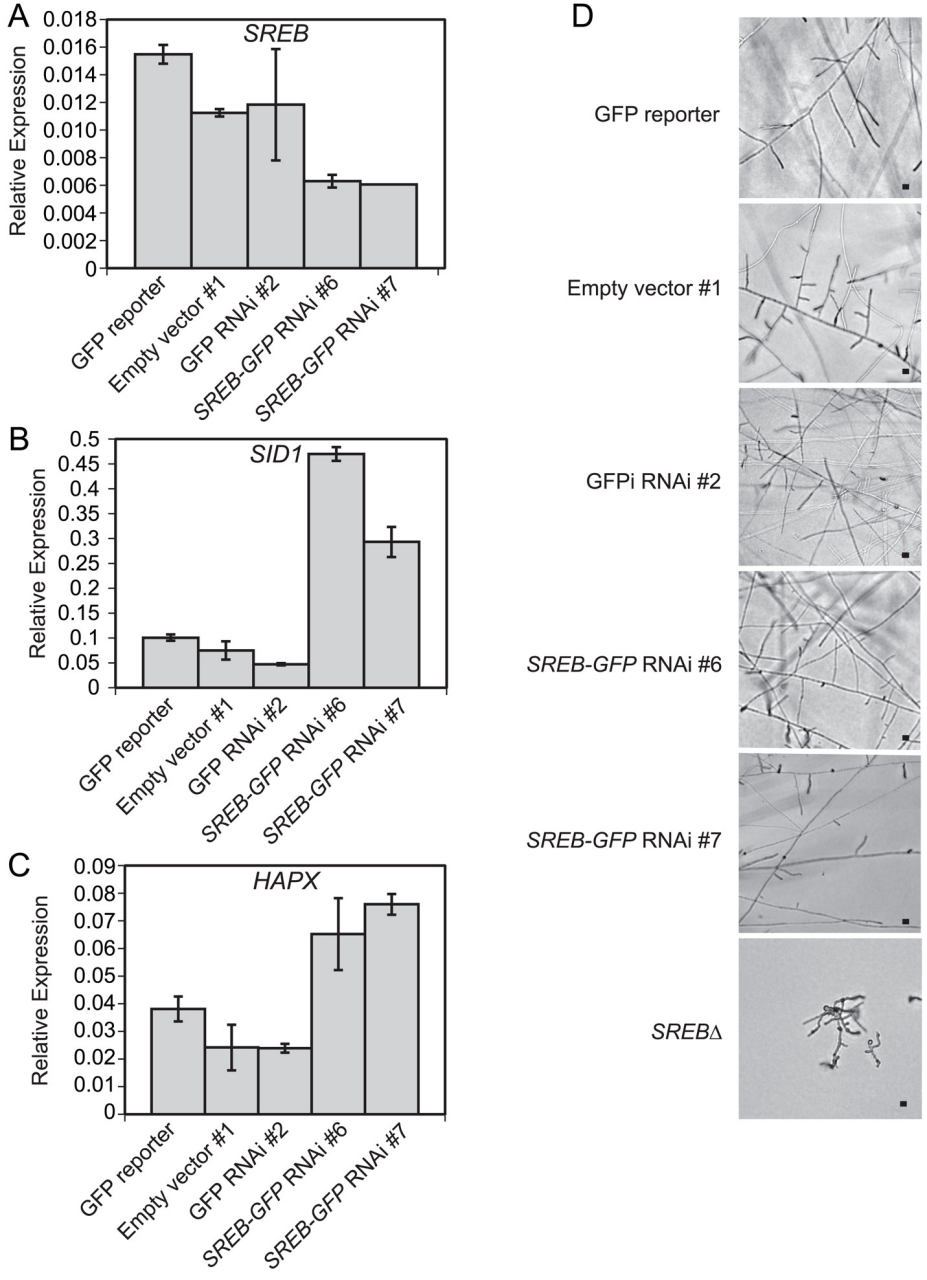
Silencing of *SREB* using RNA interference. (**A-C**) Quantitative real-time PCR analysis demonstrated a 1.8–2.5-fold decrease in *SREB* transcript and derepression of *SID1* (2.9 6.2-fold increase) and *HAPX* (1.7 3.2-fold increase) in *SREB* RNAi strains #6 and #7 compared to controls (GFP reporter, empty vector, GFP-RNAi). *SID1* encodes L-ornithine-N^5^-oxygenase, which encodes the first enzyme in siderophore biosynthesis. Quantitative RT-PCR results were averaged from 2 experiments. (**D**) *SREB-GFP* knockdown strains along with empty vector and GFP RNAi only controls converted to mycelia at 22°C. Images were photographed at 9 days incubation 22°C. Scale bar equals 10 μm.

Integration of microarray, qRT-PCR, and ChIP data suggested that *B*. *dermatitidis SREB* and *HAPX* are in a negative regulatory circuit similar to *Aspergillus nidulans* and *A*. *fumigatus* [48,49]. *B*. *dermatitidis* does not encode a homolog to *C*. *albicans SEF1*, which forms a regulatory circuit with *SFU1* and *HAP43* [50]. Moreover, deletion of *SREB* did not alter the transcription of *HAPB*, *HAPC*, or *HAPE* homologs, which form a DNA binding complex that recruits *HAPX* to CCAAT promoter sites of target genes [48]. *HAPX* transcript abundance was substantially higher in the *OE* strains (15.5–53.1-fold) than *SREB*∆ (1.7–3.5-fold) compared to WT (**Figs 4A** and **10B**). To investigate if less extreme changes in transcript abundance for either *HAPX* or *SREB* affected the phase transition, *SREB* was targeted for gene silencing using a GFP sentinel RNAi system [51]. *SREB-GFP* silenced strains had a 1.8–2.5-fold reduction in transcript abundance compared to controls (GFP reporter, empty vector, GFP-only silenced) (**Fig 11A**). *SREB-GFP* RNAi strains had increased *SID1* transcript abundance under iron-replete (10 um FeSO4) conditions compared to controls (**Fig 11B**). *HAPX* transcript was increased 1.7–3.2-fold in *SREB* RNAi versus controls (**Fig 11C**). Following a drop in temperature, *SREB-GFP* silenced strains converted to mold at 22°C (**Fig 11D**). Moreover, there was no delay in the phase transition. This data suggested that the 1.7–3.5-fold increase in *HAPX* transcript in *SREB*∆ was not responsible for the defect in phase transition at 22°C; larger changes in *HAPX* transcript abundance are required to impact morphologic development at 22°C.

## Discussion

We used whole genome gene expression microarrays and bioinformatics analyses to identify and characterize the *SREB* regulon in *B*. *dermatitidis* following a drop in temperature from 37°C to 22°C. The microarray experiments captured the transcriptional response early in the phase transition (0 48-hrs) and under conditions (10 μM FeSO_4_) that promote binding of SREB to DNA. This facilitated analysis for how SREB affected disparate processes including the morphologic switch and iron assimilation; prior research demonstrated exogenous iron neither suppressed nor enhanced the defect in phase transition observed for *SREB*∆ [18]. Further delineation of the *SREB* regulon was accomplished using ChIP-qPCR integrated with microarray and MAST analyses (along with ChIP-seq) to identify a subset of genes directly bound and regulated by *SREB in vivo*. This included genes involved with the regulation of siderophore biosynthesis and uptake, iron homeostasis, and genes unrelated to iron assimilation. The results herein represent the first genome-wide transcriptional analysis integrated with *in vivo* transcription factor binding data during the transition from yeast to mold in *B*. *dermatitidis*. Functional analysis of *SREB*∆ suggested that alterations in lipid metabolism contribute to the defect in the phase transition. In addition, chromatin immunoprecipitation data along with RNAi and overexpression analyses indicated that *SREB* was in a regulatory circuit with *HAPX*. Alteration of this circuit by either deletion of *SREB* or strong overexpression of *HAPX* impaired the conversion to mold at 22°C.

Analysis of the microarray data demonstrated *SREB* had pleiotropic effects on transcription during growth as yeast at 37°C and during the phase transition following a drop in temperature to 22°C. Deletion of *SREB* affected the transcription of 11.6–19.9% of genes (1109–1910 / 9,583) across the time course. Similarly, deletion of *CIR1*, a homolog of *SREB* important for thermotolerance at 37°C, impacted transcription for 21.0% of genes (1,623 / 7,738) in the *C*. *neoformans* genome under iron-replete conditions [24]. RNA interference targeting *SRE1* in *H*. *capsulatum*, which also affects the conversion from yeast to mycelia, altered transcription of 9.6% genes (364 / 3810) [19]. In contrast, deletion of *SREA* in *A*. *fumigatus* and *SFU1* in *Candida albicans* altered transcription for 0.6% (49 / 8975) and 2.0% (139 / 7116) genes when iron was abundant, respectively [52,53]. Unlike *CIR1*∆ or *SRE1* knockdowns, *SREA*∆ mutants are viable at 37°C and *SFU1*∆ mutants convert to hyphae under inducing conditions [52,53]. A shared feature among these GATA transcription factors is the regulation of genes involved with iron assimilation [19,24,52,53]. However, stark differences in transcriptional regulation exist between GATA transcription factors important for temperature adaptation and those that do not affect thermotolerance or morphology: *SREB*, *SRE1*, and *CIR1* affect the transcription of genes involved with diverse processes, whereas *SREA* and *SFU1* regulate a limited set of genes.

Similar to *SREB*, the transcription factors encoded by *SRE1*, *CIR1*, *SREA*, and *SFU1* can either induce or repress gene transcription [19,24,52,53]. For *CIR1*∆ and *SREB*∆ yeast, the ratio of genes with increased to decreased transcription was slightly greater than 1 [24]; at 22°C, the ratio for DE genes in *SREB*∆ was close to 1. In contrast, the majority of DE genes in *A*. *fumigatus SREA*∆ were increased [52]. In *C*. *albicans*, conflicting results have been obtained from different *SFU1*∆ strains [50,53]. To further illuminate how deletion of *SREB* affected transcription during the phase transition, WGCNA was performed to identify groups of co-expressed genes. This demonstrated that deletion of *SREB* affected transcription differently among co-expressed gene sets. This included subsets of DE genes in *SREB*∆ that failed to properly increase or decrease following a drop in temperature as well as genes with reduced transcript abundance at 37°C and 22°C.

GO analysis offered additional support that deletion of *SREB* affected the transcription of genes with diverse functions. Analysis of DE genes identified 17 GO terms enriched in *SREB*∆ ranging from iron ion binding, catalytic activity, fatty acid biosynthetic process to carbohydrate metabolic process. Integrative analysis of genes within the enriched GO categories suggested several themes including iron ion binding, lipid biosynthesis, and amino acid metabolism. Genes that encoded iron ion binding proteins were involved with oxidative stress response and lipid metabolism. The absence of a coordinated or sustained transcriptional pattern for catalase isoforms and superoxide dismutase suggested that *SREB*∆ cells were not under substantial iron-related oxidative stress. Moreover, prior research demonstrated that exogenous iron concentrations did not affect the defect in the morphologic switch in *SREB*∆ [18]. GO enrichment performed by Jung and colleagues on *CIR1*∆ and our analysis of *SREB*∆ indicated involvement of GATA transcription factors with lipid metabolism; *CIR1*∆ was enriched for fatty acid metabolism (GO:0006631) and *SREB*∆ for fatty acid biosynthetic process (GO:0006633) [24]. In addition to altering lipid metabolism, deletion of *SREB* affected genes involved with the biosynthesis of 16 amino acids. Strikingly, the majority of DE genes had reduced transcript abundance at 24 and 48-hrs 22°C. This finding may reflect the distinct morphologic differences between *SREB*∆ and WT at these time points. *B*. *dermatitidis* and *H*. *capsulatum* amino acid concentrations are approximately 3-fold higher in mycelia than yeast [54,55].

Genes involved with carbohydrate metabolism, including hydrolysis of O-glycosyl linkages, were enriched at 24 and 48-hrs 22°C. Three patterns were observed: increased transcription of chitinases, reduced transcription of glucanases, and no impact on biosynthetic genes for alpha or beta-glucan. Homologs of these genes in *S*. *cerevisiae* are involved with cell wall remodeling or maintenance. The impact of altered cell wall remodeling on *SREB*∆ at 22°C was likely limited because the addition of exogenous saturated fatty acids was able to accelerate filamentous growth at 22°C. Similarly, the effect of reduced transcription of *BDBG_00734*, a homolog of *S*. *cerevisiae GAS5*, which is bound and regulated by SREB at 37°C had limited impact; prior research demonstrated similar growth rate of *SREB*∆ and WT as yeast [18].

One of the major functions of *SREB* was to regulate iron homeostasis by repressing the transcription of genes important for the biosynthesis and transport of siderophores when iron was abundant. Genes predicted to be involved with the biosynthesis of secreted siderophores were located in a gene cluster, whereas biosynthetic genes for ferricrocin, an intracellular storage siderophore, were not clustered. This arrangement is conserved in other fungi including *H*. *capsulatum* and *A*. *fumigatus* [29,56]. Using ChIP-qPCR and ChIP-seq, we demonstrated *in vivo* that *SREB* bound upstream of clustered (e.g., *SID1*, *NRPS*, *ACE1*) and non-clustered (e.g., *SIDC*) siderophore biosynthetic genes. For genes in the SBGC, SREB bound regions with extended GATA motifs (ATC-w-gAta-a) as predicted by gel electrophoretic-mobility shift [29] and *GFP* reporter [19] assays. We extended these findings by demonstrating *in vivo* that SREB binds the promoter *SIDC*, which has the classic GATA motif, A-GATA-A. Thus, SREB regulates extracellular and intracellular siderophore biosynthetic gene transcription by binding to regions with extended and classic GATA motifs.

Reductive iron assimilation (RIA) serves as a primary mechanism for iron uptake in *C*. *neoformans*, *C*. *albicans*, and *S*. *cerevisiae*, which do not produce siderophores [24,38]. Some fungi such as *Schizosaccharomyces pombe* can synthesize siderophores and use RIA to acquire iron [57]. The tripartite machinery for RIA consists of a ferric reductase (e.g. *FRE1*), ferrous permease (e.g. *FTR1*), and multicopper oxidase (e.g. *FET3*) [39]. Despite encoding for a putative ferric reductase and a multicopper oxidase, which are under the transcriptional control of SREB, homologs to *FTR1*/*FTRA* are absent in the sequenced *B*. *dermatitidis* genomes (SLH14081, ER-3, 18188, 18187, 26199). Similarly, the genomes of other closely related dimorphic fungi did not encode *FTR1/FTRA* homologs; however, some *H*. *capsulatum* genomes were predicted to contain *FTR1/FTRA* homologs. Collectively, this suggests that ferrous transport for RIA in *B*. *dermatitidis* occurs either through an unrecognized transmembrane transporter such as *ZFT* (BDBG_05224) or that RIA is dispensable. Moreover, *B*. *dermatitidis* has an RIA-independent mechanism for acquiring iron by secreting a glutathione-dependent ferric reductase (GGT1) [58]. In *H*. *capsulatum*, GGT1-mediated ferric reduction is important for virulence [40]. Unlike siderophore-mediated iron uptake, microarray analysis indicated that *GGT1* transcription was not under the control of *SREB* at 37°C. This indicated *SREB* regulates some, but not all mechanisms for iron assimilation in *B*. *dermatitidis*.

The morphologic switch between yeast and mold in *B*. *dermatitidis*, *H*. *capsulatum*, and *Paracoccidioides brasiliensis* is associated with changes in lipid concentration. Lipid content is often ≥ 2-fold higher in mycelia than yeast [59,60]. Microarray and quantitative lipid analyses demonstrated that deletion of *SREB* reduced TAG and ergosterol concentrations, but had limited effect on phospholipids and free fatty acids (except for an increase in stearic acid at 48-hrs 22°C). As the time course progressed, the quantitative differences for ergosterol and TAG between *SREB*∆ and WT became more pronounced, which likely reflected the increased number of genes in these pathways that had reduced transcript abundance at 22°C versus 37°C. ChIP-qPCR and ChIP-seq analyses suggested that *SREB* had direct and indirect effects on genes involved with the biosynthesis of neutral lipids (TAG, ergosterol).

The decrease of neutral lipids was likely responsible for the reduction in LDs at 22°C in *SREB*∆; hydrolases involved with LD breakdown were not upregulated. Lipid droplet formation in eukaryotes including fungi is linked to TAG content as well as fatty acids, which serve as a substrate for TAG and sterol ester biosynthesis [44,45,61–63]. In *S*. *cerevisiae*, the biosynthesis of TAG contributes to lipid droplet formation to a greater degree than the biosynthesis of sterol esters [63]. The addition of exogenous saturated fatty acids (16:0 or 18:0) to *SREB*∆ transiently restored LD formation and accelerated the phase transition at 22°C. Importantly, exogenous 16:0 and 18:0 did not increase LD content or accelerate the conversion to hyphae in WT cells. The increase in LD content at 24-hrs followed by a decrease at 48-hrs 22°C in *SREB*∆ suggested that LDs are biosynthesized and degraded during the phase transition. The presence of LDs during filamentous growth in *SREB*∆ cells treated with 16:0 or 18:0 indicated that the loss of LDs in untreated cells was due to a reduction in biosynthesis and not from impaired intracellular LD transport. Thus, deletion of *SREB* disrupted LD homeostasis at 22°C in which reduced biosynthesis could not meet catabolic demands during filamentous growth. TAG and sterol esters in LDs can serve as a source of carbon for energy and lipids for membrane biosynthesis [64]. The increase in stearic acid (18:0) at 48-hrs 22°C in *SREB*∆ may represent a compensatory response to altered LD biosynthesis; however, it was not enough to overcome reduced concentrations of neutral lipids. Collectively, these data suggest that lipid metabolism including LD homeostasis may contribute to the morphologic switch from yeast to mold following a drop in temperature to 22°C.

Prior research on saturated and unsaturated fatty acids in the dimorphic fungi has focused on the heat shock response and thermotolerance of *H*. *capsulatum* during the transition to yeast following an increase in temperature to 37°C [65,66]. The addition of saturated fatty acids rescued the heat shock response by increasing *HSP82* transcription in a temperature sensitive strain, whereas unsaturated fatty acids blunted the transcription of *HSP82* in response to elevated temperature in a thermotolerant strain [66]. For *SREB*∆, the addition of saturated fatty acids enhanced the morphologic shift following a drop in temperature to 22°C, whereas unsaturated fatty acids did not impact the phase transition. Taken together, these data suggest that fatty acids and lipid metabolism influence the response to temperature at 37°C and 22°C.

*B*. *dermatitidis SREB* and *HAPX* likely form a negative regulatory circuit similar to *A*. *fumigatus* and *A*. *nidulans*. Functional analysis demonstrated a novel role for this circuit in *B*. *dermatitidis*. Deletion of *SREB* or overexpression of *HAPX* impaired the temperature-dependent morphologic switch. In *A*. *fumigatus* and *A*. *nidulans*, the *SREA-HAPX* circuit governs the cellular response to exogenous iron with SREA repressing *HAPX* transcription when iron is abundant, and HAPX inhibiting *SREA* transcription under iron poor conditions [48,49]. In *C*. *albicans*, the regulatory circuit involving *HAP43*, *SFU1*, and *SEF1* mediates the ability of this pathogen to adapt to niches within the mammalian host that have radically different iron concentrations [46 **,** 67,68]. Similar to *A*. *fumigatus*, *A*. *nidulans*, and *C*. *albicans*, transcript abundance of *B*. *dermatitidis HAPX* and *SREB* is influenced by the presence or absence of exogenous iron [18]. In the broadest sense, the *SREA*-*HAPX* and *SFU1*-*SEF1*-*HAP43* regulatory circuits mediate adaptation to changing environmental conditions. In addition to responding to exogenous iron, *B*. *dermatitidis SREB* and *HAPX* influence morphogenesis at 22°C. The defect in the phase transition in *SREB*∆ is unlikely to be due to *HAPX* derepression because *SREB* knockdown strains, which had similar levels of *HAPX* transcript as *SREB*∆, converted to mold. Moreover, the morphologic defect in *HAPX* OE strains was unlikely to be mediated through *SREB* because its repression was transient. These findings suggest that the amount of *SREB* and *HAPX* transcript influenced the morphologic response to ambient temperature.

In summary, we characterized the *SREB* regulon in *B*. *dermatitidis* during growth as yeast at 37°C and during the early part of the phase transition at 22°C. Deletion of *SREB* affected the transcription of genes involved with iron homeostasis, iron ion binding, amino acid biosynthesis, carbohydrate metabolism, lipid biosynthesis, and the transcriptional regulator *HAPX*. Moreover, deletion of *SREB* altered transcript differently among subsets of DE genes including those that failed to properly increase or decrease following a shift in temperature to 22°C. Integration of gene expression microarray and chromatin immunoprecipitation studies demonstrated that *SREB* binds and regulates genes involved with siderophore biosynthesis and iron uptake as well as genes unrelated to iron assimilation. *SREB*∆ cells had reduced TAG and ergosterol concentration along with decreased numbers of lipid droplets at 22°C. Moreover, experimental analysis suggested that LDs are biosynthesized and degraded during the phase transition and that lipid metabolism may contribute to filamentous growth at 22°C. Exogenous palmitic (16:0) and stearic (18:0) accelerated the morphologic shift and transiently restored LD formation in *SREB*∆ at 22°C. Deletion of *SREB* resulted in derepression of *HAPX* and functional analysis suggested that *SREB* and *HAPX* are in a negative regulatory circuit. Both *SREB* and *HAPX* affected the morphogenesis of *B*. *dermatitidis* at 22°C; however, large changes in transcript abundance by gene deletion for *SREB* or strong overexpression for *HAPX* were required.

## Methods

### Strains and media

*B*. *dermatitidis* strains used included American Type Culture Collection (ATCC) strain 26199, *SREB*∆, SREB-3xHA, and HAPX-OE. *B*. *dermatitidis* 26199 was originally isolated from a patient from South Carolina and is virulent in a murine model of pulmonary infection (ATCC 26199 has been used as a standard laboratory strain since the 1970’s and was a gift from the laboratory of Bruce S. Klein. This strain was not isolated from a patient for this specific study) [69–71]. *SREB*∆ was generated by targeted deletion of *SREB* in strain 26199 [18]. SREB-3xHA was engineered to contain an in-frame 3x-hemagglutinin (HA) epitope tag at the C-terminus in strain 26199. HAPX-OE strains were engineered to overexpress *HAPX* in strain 26199. *B*. *dermatitidis* cultures were maintained as yeast on *Histoplasma* macrophage medium (HMM) at 37°C containing 3 μM or 10 μM FeSO_4_ with or without hygromycin 25 μg ml^-1^ [18,72]. Cultures grown in liquid HMM at 37°C or 22°C were supplemented with 0, 1, 5, or 10 μM FeSO_4_. F-12 Ham’s nutrient mixture lacking FeSO_4_ was used for preparation of HMM. For iron chelation experiments, deferiprone (3-hydroxy-1,2-dimethyl-4(1H)-pyridone; Sigma-Aldrich, Saint Louis, MO) 0.5 mM or 1.0 mM was added to liquid HMM supplemented with 1, 5, or 10 μM FeSO_4_. *Agrobacterium tumefaciens* strain LBA1100 harboring the Ti helper plasmid pAL1100 (gift from C. van den Hondel; Leiden University, the Netherlands) was maintained on Luria-Bertani (LB) medium supplemented with 0.1% glucose, spectinomycin 100 ug/ml, and kanamycin 100 ug/ml after transformation with a binary vector [18,73].

### Quantification of cell morphology and viability

To quantify the percentage of cells with yeast morphology, germ tube development, and hyphal growth, cells were stained with calcofluor white (Sigma Aldrich) [74]. Calcofluor white staining facilitated differentiation of germ tubes and hyphae by the absence or presence of septae, respectively. Eosin exclusion staining was performed to assess cell viability [18].

### Whole genome gene expression microarrays

To compare transcription between *SREB*∆ and an isogenic WT isolate, 385K whole genome gene expression microarrays (Roche NimbleGen, Inc., Madison, WI) were custom designed for *B*. *dermatitidis* using the annotated sequence of strain SLH14081 (9,587 genes; www.broadinstitute.org). A total of 9,583 genes (nuclear and mitochondrial) were represented on each array in quadruplicate with 9,571 genes having a probe-set comprised of 10 unique oligonucleotide probes; 12 genes had less than 10 unique oligonucleotide probes. Oligonucleotide probes with sufficient specificity were unable to be designed for a mitochondrial laglidadg endonuclease (BDBG_17014), mitochondrial ATP synthase F0 subunit 8 (BDBG_17017), mitochondrial NADH dehydrogenase subunit 5 (BDBG_17032), and a predicted protein (BDBG_03106). Empty features on the array were filled with randomly generated probes with similar length and G+C content as the NimbleGen internal controls.

### Experimental conditions and isolation of RNA for microarray analysis

Total RNA was isolated from 3 biological replicates of *SREB*∆ and the isogenic wild-type control at 37°C and 6 hours, 24 hours, and 48 hours following a drop in temperature to 22°C (24 total samples). All cultures were grown in liquid HMM under iron replete conditions (10 μM FeSO_4_). All glassware was pre-treated with 2N HCl and extensively washed with ddH_2_O. Prior investigation of *SREB* demonstrated that transcription was induced in the presence of exogenous iron [18]. In addition, optimal binding of SREB homologs to DNA requires coordination of iron by its cysteine-rich region [29,75]. The phenol-guanidinium thiocyanate-1-bromo-3-chloropropane method was used to extract total RNA [76]. In brief, *B*. *dermatitidis* cells were washed with PBS (pre-warmed to 37°C or 22°C), frozen in liquid nitrogen, lysed by grinding in a mortar and pestle, and treated with TRI Reagent followed by 1-bromo-3-chloropropane (Molecular Research Center Inc., Cincinnati, OH). Total RNA was precipitated using a 1:1 concentration of isopropanol and high salt solution (Molecular Research Center, Inc., Cincinnati, OH), and washed with 75% ethanol. Following resuspension in water pre-treated with diethyl pyrocarbonate (DEPC; Calbiochem, San Diego, CA), total RNA was treated with turboDNAase (Applied Biosystems/Ambion, Austin Texas), and purified using an RNeasy kit (Qiagen, Valencia, CA). To optimally remove guanidine salts, an extra wash of RPE buffer was performed during RNAeasy purification. RNA quality was assessed using an Agilent bioanalyzer (Agilent Technoligies, Santa Clara, CA). Double-stranded cDNA was synthesized and purified at the Gene Expression Center at the University of Wisconsin–Madison. Roche NimbleGen performed Cy3 labeling, hybridization, scanning, and RMA (Robust Multi-Array analysis) normalization for all 24 microarrays at their facility in Reykjavík, Iceland.

### Statistical analysis of microarray data

To identify differentially expressed genes in *SREB*∆ versus WT at each time point, two different statistical programs were used, LIMMA (Linear Models for Microarray data) and EBarrays (Empirical Bayes Methods for Microarrays) [30 **,** 31]. Both programs were executed using R-based software. LIMMA used a Bayesian approach to fit fluorescent intensity data for all genes across the different arrays into a linear model, which was followed by analysis for differential gene expression between *SREB*∆ and WT using a moderated *t*-statistic [30]. We used q-values to adjust Limma results in order to remain at a 5% false discovery rate [77]. EBarrays used fluorescent intensity for all genes across all microarrays to model the posterior probability of a single gene to determine whether it had equivalent (*µ*_1_ = *µ*_2_) or different expression (*µ*_1_ *µ*_2_) in *SREB*∆ versus WT [31]. A lognormal-normal model with moderated variance was used in the EBarrays analysis because there were 3 biological replicates for *SREB*∆ and WT at each time point. An individual gene was considered differentially expressed if statistical significance was met for both LIMMA (q-value < 0.05) and EBarrays (posterior probability > 0.95) analyses. Heat maps were generated using CIMminer from the Genomics and Bioinformatics Group (Laboratory of Molecular Pharmacology, Center for Cancer Research, NCI, NIH, DHHS) (http://discover.nci.nih.gov). Gene expression microarray data have been deposited to the Gene Expression Omnibus (http://www.ncbi.nlm.nih.gov/geo/) (accession number: GSE67724).

### Gene ontology (GO) annotation and enrichment analysis

GO terms were assigned to the proteome encoded by the *B*. *dermatitidis* genome using InterProScan [78]. The annotation file contained the gene identification number with the associated GO term and GO identification number. Fisher’s exact test was used to test whether the number of DE genes as determined by EBarrays (posterior probability > 0.95) was higher for genes inside the GO category than outside the GO category. Q-values were calculated from the Fisher p-values in order to remain at a 5% false discovery rate [77]. The enrichment test suggests functionality of a group of genes that are differentially expressed under a particular condition.

### Weighted gene co-expression network analysis (WGCNA)

We used WGCNA [79] to find clusters (or modules) of highly correlated genes, and for creating a module eigengene to relate modules to one another or to a clinical trait. In the first step of the analysis, we calculated a correlation matrix for the genes, and raised it to a power (β = 12) to create an adjacency matrix of connection strength. The sum of the connection strengths with the other network genes was defined as connectivity, and a topological overlap measure was calculated based on the number of shared neighbors. The dendrogram was created by hierarchical clustering 1 minus the topological overlap, and branches of the tree were cut using a dynamic tree cut algorithm to define modules [80]. Module eigengenes are the first principal component of a module, and can also be thought of as the average gene expression for all genes in a module for a sample. We used *allez* to determine GO and KEGG enrichment in each module, and calculated correlation between module eigengenes and time points.

### Extraction of ferricrocin and reverse-phase high-pressure liquid chromatography (HPLC)

*SREB*∆ and WT yeast were grown in liquid HMM supplemented with 10 μM FeSO4 at 37°C. Once cells reached stationary growth when measured by spectrophotometric absorbance at 600 nm, they were washed 3 times in 1x phosphate buffered saline (PBS) to remove extracellular siderophores from the medium, flash frozen in liquid nitrogen, ground in a mortar and pestle, resuspended in PBS, and centrifuged to pellet the cellular debris. The supernatant was collected, treated with 2% ferric chloride, and applied to a column packed with Amberlite XAD-2 resin (Supelco, Bellefonte, PA). Resin and column preparation along with siderophore isolation was performed as previously described [18]. Following XAD-2 chromatographic isolation, siderophores were separated on a C18 column (Agilent Eclipse XDB-C18 column; 4.6x150 mm) using a water-acetonitrile gradient containing 0.1% trifluoroacetic acid (Sigma-Aldrich). The gradient of acetonitrile was increased from 5% to 15% over 10 minutes, then to 25% over 22 minutes. Flow rate was 0.75 ml/min and absorbance was measured at 465 nm. Retention times were compared to a ferricrocin siderophore standard (HPLC calibration kit–ferrichromes; EMC microcollections, Tubingen, Germany).

### Triphenyltetrazolium chloride (TTC) overlay agar assay

Wild type and *SREB*∆ were incubated (5 x 10^5^ cells) on iron poor HMM (IPM) and iron-replete (10 μM FeCl_3_) HMM media supplemented with 50 μM CuSO_4_ at 37°C for 4 days prior to exposure to 0.1% triphenyltetrazolium chloride (TTC; Sigma-Aldrich) [81,82]. TTC as added to overlay agar (1.5% bactoagar, 0.067 M phosphate buffer, pH 7) after autoclaving.

### Isolation and characterization of lipids

*SREB*∆ and WT were grown at 37°C and 22°C in liquid HMM supplemented with 10 μM FeSO_4_. Cells were harvested, washed with H_2_O twice, resuspended in 0.97% KCl, and beaten with beads prewashed with methanol-chloroform (1:1). Lipids were extracted using chloroform-methanol (2:1) containing 5mg/L butylated hydroxytoluene (Sigma-Aldrich) in organic solvent-washed glassware (no plastics were used). Samples were mixed by vortexing for 3 minutes and incubated at 4°C for 2 hours. The samples were then treated with 0.97% KCl, vortexed for 30 seconds, and incubated at 4°C for 1 hour. Following centrifugation (300 x g for 5 minutes), the organic layer was removed and washed x 3 with 0.97% KCl. The collected lipid extracts were dried under nitrogen.

After drying, samples were reconstituted in 2:1 chloroform:methanol and lipid classes were separated by thin layer chromatography (TLC; 20 cm × 20 cm silica gel Si60 plates; Merck KGaA, Darmstadt, Germany) using heptane:isopropyl ether:acetic acid (60:40:3). After spraying TLC plates with 0.2% 2’,7’-dichlorofluorescein in ethanol, lipid classes (phospholipid, DAG, free fatty acids, TAG, sterol esters) were visualized by UV light and scraped off the TLC plate. Lipids were reextracted overnight at room temperature with chloroform: methanol (4: 1) containing 100 mg/L butylated hydroxytoluene (Sigma-Aldrich), and centrifuged 850 x g to remove silica gel particles. The internal standard, pentadecanoic acid (100 μl of 0.05 mg/ml in chloroform) was added to each sample and the organic solvents were evaporated under nitrogen. Isolated lipids were then methylated using 0.5 ml 14% boron trifluoride-methanol solution (Sigma-Aldrich), boiled for 30 minutes at 100°C, cooled, mixed with 1 ml hexane, vortexed for 30 seconds, mixed with 0.5 ml H_2_O and centrifuged (850 x g for 5 minutes) at room temperature. The top hexane layer, which contained methyl ester lipid derivatives, was transferred to a new glass vial, dried under nitrogen, resuspended in 100 μl hexane, and transferred to gas chromatography vials.

Fatty acid methyl esters were identified by gas chromatography using a Hewlett-Packard 6890 (Hewlett Packard, Palo Alto, CA) equipped with a capillary column coated with DB-225 (30 m × 0.25 mm, internal diameter of 0.25 μm) (Agilent Technologies Inc., Wilmington, DE). Column temperature was kept at 70°C for 1 min, increased to 180°C at a rate of 20°C/min and then to 220°C at a rate of 3°C/min. The temperature was kept at 220°C for 15 min. The front inlet was set at 250ºC and the detector temperature was set at 300ºC. Peaks were identified by a comparison of retention times with a set of authentic FA standards (Supelco, Bellefonte, PA). The abundance of FAs was calculated from the relative peak areas [83]. The fatty acid abundance reported reflects the fatty acids present at the end of the protocol.

Sterols were extracted and analyzed according to the protocol described by Zarnowski et al. [84]. Briefly, sterols were reextracted by direct saponification with 1 ml of 6% (w/v) KOH in methanol for 1 h at 90°C. After addition of 1 vol of H_2_O, the unsaponifiable fraction was extracted three times with 3 volumes of C_6_H_6_. The sterols were identified by FAST GC-MS and quantified by the peak area ratio of sterol/internal standard (cholesterol) in the GC-FID chromatogram. The characterization of sterol was performed on a Shimadzu 2010 Plus system equipped with a Shimadzu QP 2010 Ultra mass spectrometer detector. The separation was performed on a Zebron ZB-5MS capillary column (10 m × 0.1 mm i.d., 0.1 μm film thickness, Phenomemex). The system was operated in the constant linear velocity (70 cm/s) using helium as a carrier gas and the sample was injected in the split mode (Split ratio 20:1). The GC oven temperature was started at 80 °C, increased to 220 °C at a rate of 60 °C min^−1^, then to 300 °C at a rate of 20 °C min^−1^ and finally held to 300 °C for 1.5 min. The injection port, transfer line and ion source temperature were maintained at 285, 300 and 250 °C, respectively. The MS was run in the mode of electron impact (EI) mode with an electron energy of 70 eV and the spectra were scanned in the range of m/z 50–500. The sterols were identified by comparing their mass spectra with the standard mass spectra in the NIST MS library. Additional FID detector was allowed the quantitative analysis of sterol. The FID temperature was 320 °C and the oven temperature program was the same as to the GC–MS operation.

### Antifungal drug susceptibility testing

*B*. *dermatitidis SREB*∆ and WT yeast (1.2 x 10^6^) were uniformly spread on 6-well plates (Corning Inc., Corning, NY) containing HMM supplemented with 10 μM FeSO_4_. Antibiotic assay paper discs (6 mm; Whatmann/GE Healthcare Life Sciences, Pittsburgh, PA) were impregnated with 0–10 μg Amphotericin B deoxycholate dissolved in dimethyl sulfoxide (DMSO; Sigma-Aldrich) or 0–1 μg voriconazole dissolved in water (Pfizer, New York, NY), placed in the center of each well, overlaid with 0.5% agarose top agar, and incubated at 37°C. Antifungal susceptibility was assessed by the diameter of the clear zone surrounding the discs impregnated with amphotericin B deoxycholate or voriconazole.

### Lipid droplet staining and fatty acid supplementation

Lipid droplets in *SREB*∆ and WT cells were stained with BODIPY 493/503 (Life Technologies, Carlsbad, CA) at 37°C and 22°C [43]. Cultures were grown in liquid HMM supplemented with 10 μM FeSO_4_, washed in 1x PBS, and fixed in 2% paraformaldehyde to optimize intracellular entry of BODIPY 493/503. Following fixation, cells were washed and resuspended in 1x PBS. Yeast cells grown at 37°C (and 6-hrs 22°C) were stained with 1:50 dilution of 0.5 mM BODIPY 493/503 and cells grown at 22°C (24-hrs and 48-hrs) were stained with a 1:25 diluation of 0.5 mM BODIPY 493/503. Optimal image capture of LDs by the microscopy camera (EXi aqua, Qimaging, Surrey, British Columbia, Canada) required a higher concentration of BODIPY 493/503 for WT and *SREB*∆ cells harvested at 24 and 48-hrs 22°C versus 37°C. Intracellular lipid droplets were visualized using fluorescent microscopy (Olympus BX60; Olympus America, Center Valley, PA) using an excitation filter with a range of 460–490 nm. ImageJ was used to facilitate quantitative LD analysis.

*SREB*∆ and WT cells grown in iron-replete (10 μM FeSO_4_) liquid HMM at 37°C were supplemented with 0.5–1.0 mM palmitic acid (16:0), palmitoleic acid (16:1n7), stearic acid (18:0), and oleic acid (18:1n9) (Cayman Chemical, Ann Arbor, MI) prior to incubation at 22°C. Palmitic and stearic acid were nearly insoluble in aqueous solutions and required solubilization in DMSO (Sigma-Aldrich) prior to addition to iron-replete liquid HMM (final DMSO concentration was 1.43%). Palmitoleic and oleic acid were soluble in liquid HMM. *SREB*∆ and WT cells treated with fatty acids along with controls (untreated, DMSO only) were harvested at 37°C and 6, 24, and 48-hrs at 22°C. Harvested cells were washed twice with 1x PBS, fixed in 2% paraformaldehyde, and stained with either BODIPY 493/503 or calcofluor for analysis of LDs and morphology, respectively.

### Chromatin immunoprecipitation with quantitative real-time PCR (ChIP-qPCR)

To perform ChIP, we engineered SREB to contain a 3x-HA tag located in-frame at the C-terminus. Following PCR amplification of the *SREB* coding region using primers SREB_F1 and 3xHA_RC (**S7 Table**), the PCR product was purified using QIAquick gel extraction kit (Qiagen, Valencia, CA), sequenced by the DNA Sequencing Laboratory at the University of Wisconsin Biotechnology Center, and ligated into a vector that contained the native SREB promoter and 3’ UTR to create pBTS47-SREB-3xHA. *Agrobacterium tumefaciens*-mediated transformation was used to transform *B*. *dermatitidis* strain 26199 [73]. Transformants were selected on HMM supplemented with 25 ug/ml nourseothricin (Werner Bioagents, Jena, Germany). Western blot analysis using a ChIP-grade rabbit polyclonal HA antibody (Abcam, Cambridge, MA) was performed to assess specificity of the antibody for SREB-3xHA (**S8 Fig**). In brief, wild-type and SREB-3xHA transformed cells were beaten with beads, suspended in 1x SDS-PAGE loading buffer, boiled for 10 minutes, and loaded onto an SDS-PAGE gel (6% stacking, 10% resolving) for separation by electrophoresis. Separated proteins were transferred to Immobilon-P PVDF membrane (Millipore), probed with primary (rabbit polyclonal HA antibody; 1:1000 dilution) and secondary (1:2000 dilution of peroxidase affinipure goat anti-rabbit IgG (H+L); Jackson ImmunoResearch Laboratories, West Grove, PA), developed using ECL western blotting substrate (Pierce, Rockford, IL), and imaged using VersaDoc imaging systems (Bio-Rad). To confirm the SREB-3xHA construct was functional, *SREB*∆ was transformed with pBTS47-SREB-3xHA using *A*. *tumefaciens*-mediated DNA transfer.

For ChIP-qPCR, we modified the *Caenorhabditis elegans* protocol published by Mukhopadhyay et al. [85]. *B*. *dermatitidis* SREB-3xHA and WT cells grown in liquid HMM supplemented with 10 μM FeSO_4_ at 37°C and 22°C were treated with 1% formaldehyde (Sigma-Aldrich) for 30 minutes to crosslink HA-tagged SREB to its DNA binding site. Formaldehyde crosslinking was quenched with 0.125 M glycine for 5 minutes. Cells were washed twice in ice-cold 1x PBS, washed once in ice-cold 1x PBS containing protease inhibitor cocktail (PIC; Sigma-Aldrich), flash frozen in liquid nitrogen, ground to a fine powder using a mortar and pestle, and resuspended in FA lysis buffer containing PIC plus phenylmethylsulfonyl fluoride (PMSF). The cell lysate was centrifuged 13,600 x g for 15 minutes at 4°C, supernatant discarded, pellet resuspended in FA lysis buffer with PIC plus PMSF, and stored at -80°C. Chromatin was sheared using probe sonication (Misonix S-4000; Qsonica, Newton, CT). Cells grown at 37°C were sonicated for a total of 25 minutes of “on time”; whereas cells at 22°C were sonicated for a total of 15 minutes of “on time”. Each round of sonication consisted of 1 minute of “on time”, which was divided into 15 seconds of sonication (amplitude 30) x 4 with intervening rest periods of 30 seconds. Cells were kept ice cold during sonication and between each round of sonication, the cells were chilled for 10 seconds in a dry ice-ethanol bath to prevent overheating. From each sample, 50 μl was saved as an “input” sample and 50 μl was analyzed for efficacy of chromatin shearing by 0.8% agarose gel electrophoresis.

For ChIP, protein A agarose/salmon sperm DNA beads were prepared according to the manufacturer’s instructions (Millipore, Billerica, MA) and resuspended in FA lysis buffer containing PIC plus PMSF. Sonicated chromatin (300 μg measured by nanodrop) was pre-cleared for 1 hour at 4°C prior to adding 10 μl anti-HA along with freshly prepared beads. Following an overnight incubation at 4°C with orbital rotation, the samples were washed twice with buffer #1 (FA lysis buffer), once with buffer #2 (FA lysis buffer containing 0.5 M NaCl), once with buffer #3 (10 mM Tris pH8, 0.25M LiCl, 1 mM EDTA, 0.5% Nonidet P-40, 0.5% sodium deoxycholate), and thrice with Tris-EDTA pH 8. All washes were 3 minutes at 4°C with orbital rotation. To reverse cross-linking, ChIP’d samples were treated with proteinase K for 2 hours at 45°C, and then 65°C overnight. Input samples were treated with proteinase K at 55°C for 4 hours then 65°C overnight. DNA was isolated and purified according to the protocol in Mukhopadhyay et al., and resuspended in Tris-HCl pH 8 [85]. For qPCR, primers were designed against the GATA motif and coding region of target genes (**S7 Table**). Potential GATA motifs were identified by MAST analysis [18]. Fold enrichment was determined by calculation of ∆Ct of SREB-3xHA and WT (∆Ct = ChIP Ct–input Ct) followed by 2^-∆∆CT (∆∆CT = ∆Ct SREB-3xHA- ∆Ct WT) [85].

### ChIP with DNA sequencing (ChIP-seq)

For ChIP-seq, chromatin from yeast cells grown under iron-replete conditions (10 μM FeSO_4_) was prepared, sheared and immunoprecipitated similar to the protocol described for ChIP-qPCR; however, protein A agarose beads without salmon sperm DNA were used (otherwise the salmon sperm DNA would be sequenced in addition to *B*. *dermatitidis* DNA). Immunoprecipitated DNA (along with input DNA) from WT and SREB-3xHA strains were submitted to the University of Wisconsin Biotechnology Center for sequencing using Illumina HiSeq 2000 platform. Two biological replicates were analyzed for WT and SREB-3xHA. We aligned the ChIP and input reads using Bowtie (version 0.12.7 with parameters “-p 4-q-v 2-a—best—strata-m 2”) to the genome sequence of *Blastomyces dermatitidis* (blastomyces_dermatitidis_atcc_26199_1_supercontigs.fasta downloaded from www.broadinstitute.org/annotation/genome/blastomyces_dermatitidis/MultiDownloads.html [86–88]. We performed quality control analysis of the data using the quality metrics from Landt et al. [89]. We then used the R package mosaics in the two sample analysis with input data mode to call peaks [88]. The specific parameters of mosaics were set as follows: bin size = 150; fragment size = 150, fdr = 0.1; thres = 97.5^th^ percentile of the bin-level ChIP read count distribution. This approach identified 37 26199-specific, 847 3xHA-specific, and 242 common peaks. We further filtered these peaks by taking into account the ChIP signal at the summit. Specifically, for each peak, we considered a window size of 1000 bps around the mid-point of the original peak and defined the highest read coverage point as the summit of the peak. We further filtered peaks with less than 20 reads per one million reads at the summit. We annotated the resulting peaks by overlapping them with the 500 bps upstream of the transcription start sites using the 9180 TSS groups defined in blastomyces_dermatitidis_atcc_26199_1_transcripts.gtf (www.broadinstitute.org). ChIP-seq data have been deposited to the Gene Expression Omnibus (http://www.ncbi.nlm.nih.gov/geo/) (accession number: GSE68680).

### *B*. *dermatitidis* overexpression strains

Plasmid pLBZ1, which was a gift from Dr. Jon Woods, was used to generate pBTS47-Woods to overexpress candidate genes in *B*. *dermatitidis*. pLBZ1 contains the *H*. *capsulatum* H2B promoter and a CatB terminator with intervening *Asc*I and *Sbf*I restriction sites. The H2B promoter through the CatB terminator in pLBZ1 was amplified using forward and reverse primers containing *Cla*I and *BamH*I restriction site sequences, respectively. The amplicon was separated by 1% agarose gel electrophoresis, purified using a Qiaquick PCR purification kit (Qiagen, Valencia, CA), digested with *Cla*I and *BamH*I-HF (New England Biolabs, Ipswich, MA), and ligated into pBTS47 using T4 DNA ligase (Promega, Madison, WI) resulting in pBTS47-Woods. pBTS47 contains a nousethricin resistance cassette and was a gift from Dr. Bruce Klein. Open reading frames of candidate genes were amplified using forward and reverse primers containing *Asc*I and *Sbf*I restriction site sequences, respectively (**S7 Table**). The amplicons were separated by gel electrophoresis, purified by QiaQuick PCR purification kit, digested with *Asc*I and *Sbf*I, and ligated into pBTS47-Woods using T4 DNA ligase. *A*. *tumefaciens*-mediated DNA transfer was used to transform *B*. *dermatitidis* strain 26199 with pBTS47-Woods containing the candidate gene of interest (**S7 Table**). Transformants were selected on HMM supplemented with 25 μg/ml nourseothricin and 200 mM cefotaxime.

### Quantitative real-time PCR

Total RNA was extracted from *B*. *dermatitidis* cells grown in liquid HMM supplemented with 10 μM FeSO_4_ at 37°C and 22°C as described in the microarray section. One microgram of DNase-treated total RNA was converted to cDNA using iScript cDNA synthesis kit (Bio-Rad). Quantitative real-time PCR was performed with SsOFast EvaGreen Supermix (Bio-Rad) using a MyiQ real-time PCR detection system (Bio-Rad). Reactions were performed in triplicate using the following conditions: 1 cycle 95°C x 30 sec, followed by 40 cycles at 95°C for 5 sec, 60°C for 10 sec. Primers used for qRT-PCR are listed in S7 Table. Transcript abundance of genes involved with iron assimilation at 37°C and *HAPX* were normalized relative to the transcript abundance of GAPDH, whereas genes involved with glycerolipid biosynthesis analyzed at 37°C and 48-hrs 22°C were normalized to L34. For analyzing gene transcription at two different temperatures, 37°C and 22°C, L34 transcript abundance demonstrated the most stability (versus GAPDH and alpha-tubulin). Relative expression (RE) was calculated as RE = 2^-∆Ct^, ∆Ct = Ct_target gene_−Ct_GAPDH or L34_ [90].

## Supporting Information

S1 Table Differentially expressed genes. (XLS)

S2 Table Enriched gene ontology (GO) terms for differentially expressed genes. (XLSX)

S3 Table Gene ontology (GO) enrichment categories. (DOC)

S4 Table Shared gene ontologies for differentially expressed genes. (DOC)

S5 Table Gene locus and name for heat maps in Fig 3. (XLSX)

S6 Table Integration of ChIP-seq, ChIP-qPCR, and microarray data for genes bound and regulated by SREB at 37°C. (XLSX)

S7 Table Primer sequences. (XLSX)

S1 Fig Putative functions of differentially expressed (DE) genes within gene ontologies (GO) enriched across 22°C time points. DE genes were enriched for 4 GO terms across all 22°C time points (6-hrs, 24-hrs, 48-hrs): catalytic activity, metabolic process, oxidation-reduction process, and oxidoreductase activity. Putative function for DE genes within these broad ontologies was assigned by integration of GO term, tBLASTn, and pFAM analyses. Number of DE genes in each ontology is listed in parentheses. NOC is not otherwise categorized.
(EPS)

S2 Fig Schematic of phospholipid biosynthetic pathways. **(A)** Kennedy and cytidine diphosphate diacylglycerol (CDP-DAG) pathways are involved with the biosynthesis of phospholipids including phosphatidylethanolamine, phosphatidylcholine, phosphatidylserine, phosphatidylinositol, and cardiolipin. **(B)** Heat map of fluorescent intensity (log_2_) values of putative genes in the Kennedy and CDP-DAG pathways. DE genes are highlighted by green text. Genes numbered in (A) correspond to genes numbered in (B). Homologs of genes in *S*. *cerevisiae* are in parentheses. PIT is phosphatidyltransferase. Adjacent to the heat map, log_2_ fold change values for DE genes are shown; genes that are not DE are labeled as “—”.
(EPS)

S3 Fig Heat map of amino acid biosynthetic pathways. Heat map of log_2_ fold change values for genes involved with amino acid biosynthesis (*SREB*∆ versus wild-type). Log_2_ fold change values for DE genes are shown. Genes that are not DE are labeled as “—”.
(EPS)

S4 Fig Triphenyltetrazolium chloride assay for cell surface ferric reductase activity. Wild type and *SREB*∆ yeast (5 x 10^5^ cells) grown on iron poor HMM (IPM) and iron-replete (10 μM FeCl_3_) media were exposed to 0.1% triphenyltetrazolium chloride (TTC) for 15, 30, 60, and 90 minutes. Reduction of TTC results in the production of red pigmentation (formazan).
(TIF)

S5 Fig Ergosterol biosynthetic pathway heat map. Heat map of log_2_ fold change values for genes in the ergosterol biosynthetic pathway (*SREB*∆ versus wild-type). Differentially expressed genes are highlighted in green text. Adjacent to the heat map, log_2_ fold change values for DE genes are shown; genes that are not DE are labeled as “—”.
(EPS)

S6 Fig Antifungal drug susceptibility testing. Disc diffusion testing for amphotericin B deoxycholate (0–10 μg) and voriconazole (0–1 μg).
(TIF)

S7 Fig Morphology of cells treated with unsaturated fatty acids at 37°C and 6-hrs 22°C. (**A**) Morphology and lipid droplet straining (BODIPY 493/503) of wild type and *SREB*∆ cells treated with 0.5 mM palmitic (16:0) and stearic (18:0) acid at 37°C. Controls included untreated cells and cells grown in DMSO. Corresponding bright field microscopic images (light) are below the fluorescent images. Scale bar equals 10 μm. (**B**) Morphology and lipid droplet straining (BODIPY 493/503) of wild type and *SREB*∆ cells treated with 0.5 mM palmitic (16:0) and stearic (18:0) acid at 6-hrs 22°C. Controls included untreated cells and cells grown in DMSO. Corresponding bright field microscopic images (light) are below the fluorescent images. Black arrows indicate germ tubes. Scale bar equals 10 μm.
(TIF)

S8 Fig Morphology and viability of cells treated with oleic and palmitoleic acid. (**A**) Morphology and lipid droplet staining of *SREB*∆ untreated and *SREB*∆ 18:1n9 treated cells at 48-hrs 22°C. (**B**) WT and *SREB*∆ cells grown in iron-replete liquid HMM with or without supplementation of 0.125 mM palmitoleic acid (16:1n7) at 37°C. Growth was measured by absorbance at 600 nm. Results are averaged from 2 biological replicates from a representative experiment.
(TIF)

S9 Fig Complementation of *SREB*∆ with *SREB-3xHA*. (**A**) The morphologic defect of *SREB*∆ at 22°C was successfully complemented by the *SREB*-*3xHA* construct. *SREB*∆, empty vector, complemented strains, and wild type strains were imaged at 17 days 22°C. Scale bar is equals 10 μM. (**B**) Western blot and coomassie gel analyses of wild type 26199 and 26199 transformed with *SREB*-3xHA. Predicted molecular weight of *SREB*-*3xHA* was 71.2 kDa.
(TIF)

S10 Fig ChIP-seq enrichment for a subset of genes at 37°C involved with iron homeostasis. ChIP-seq enrichment of SREB-3xHA binding upstream of genes located in the siderophore biosynthetic gene cluster (conserved hypothetical protein, *ATRH*, *NRPS*, *SID1*, Ligase, *ACE1*) as well as genes involved with ferricrocin biosynthesis (*SIDC*), siderophore uptake (*MIRB*, *MIRC*, *SITE1*), *FRE1*, and *HAPX*. Below each tracing is the motif within the bound region along with its location with respect to the upstream start codon. Solid lines represent immunoprecipitated chromatin for the control (26199 WT) and SREB-3xHA. Dashed lines represent the input control. Tracings are from two biological replicates, A and B.
(EPS)

S11 Fig ChIP-seq enrichment for a subset of genes at 37°C unrelated to iron assimilation. ChIP-seq enrichment of SREB-3xHA binding upstream of genes encoding a WD repeat protein, cation diffusion facilitator, GNAT family acetyltransferase, thioesterase, glycolipid surface protein, C-5 sterol desaturase, and an amino acid acetyltransferase. Below each tracing is the motif within the bound region along with its location with respect to the upstream start codon. Solid lines represent immunoprecipitated chromatin for the control (26199 WT) and SREB-3xHA. Dashed lines represent the input control. Tracings are from two biological replicates, A and B.
(EPS)
