# Supplementary material for: Fungal Morphology, Iron Homeostasis, and Lipid Metabolism Regulated by a GATA Transcription Factor in Blastomyces dermatitidis
Source: PLoS Pathog. 2015 Jun 26;11(6):e1004959. doi: 10.1371/journal.ppat.1004959 (PMC4482641; doi:10.1371/journal.ppat.1004959)
Supplement: S3 Table — (DOC) [file ppat.1004959.s003.doc]

**S3 Table. Gene ontology (GO) enrichment categories**

| **GO term** | **GO ID** | **Number of genes within the ontology** | **Number of differentially expressed (DE) genes within the ontology*** |
| --- | --- | --- | --- |
| **Ontology: molecular function** |  |  |  |
| Iron ion binding | GO:0005506 | 89 | 50 (56.2%) |
| Catalytic activity | GO:0003824 | 642 | 321 (50.0%) |
| Oxidoreductase activity | GO:0016491 | 254 | 157 (61.8%) |
| FAD binding | GO:0050660 | 73 | 40 (54.8%) |
| Amino acid transmembrane  transporter activity | GO:0015171 | 24 | 18 (75%) |
| Hydrolase activity, hydrolyzing  O-glycosyl compounds | GO:0004553 | 41 | 29 (70.7%) |
| Transporter activity | GO:0005215 | 57 | 33 (57.9%) |
| **Ontology: biologic process** |  |  |  |
| Transmembrane transport | GO:0055085 | 276 | 161 (58.3%) |
| Metabolic process | GO:0008152 | 591 | 314 (53.1%) |
| Oxidation reduction process | GO:0055114 | 377 | 187 (49.6%) |
| Fatty acid biosynthetic process | GO:0006633 | 18 | 14 (77.7%) |
| Amino acid transport | GO:0006865 | 24 | 18 (75%) |
| Transport | GO:0006810 | 158 | 80 (50.6%) |
| Carbohydrate metabolic process | GO:0005975 | 86 | 45 (52.3%) |
| **Ontology: Cellular Component** |  |  |  |
| Integral to membrane | GO:0016021 | 220 | 113 (51.4%) |
| Membrane | GO:0016020 | 293 | 136 (46.4%) |
| Endoplasmic reticulum | GO:0005783 | 20 | 13 (65%) |

GO ID refers to gene ontology identification number.

*DE by EBarrays
