# Supplementary material for: Fungal Morphology, Iron Homeostasis, and Lipid Metabolism Regulated by a GATA Transcription Factor in Blastomyces dermatitidis
Source: PLoS Pathog. 2015 Jun 26;11(6):e1004959. doi: 10.1371/journal.ppat.1004959 (PMC4482641; doi:10.1371/journal.ppat.1004959)
Supplement: S4 Table — (DOC) [file ppat.1004959.s004.doc]

**S4 Table. Shared gene ontologies for differentially expressed genes**

|  | **Percentage of genes with shared GO terms** | | | |
| --- | --- | --- | --- | --- |
| **Gene Ontology** | **Catalytic activity** | **Metabolic process** | **Oxidation-reduction** | **Oxidoreductase activity** |
| Catalytic activity (321) | 100% (321/321) | 66.6% (214/321) | 23.7% (76/321) | 23.1% (74/321) |
| Metabolic process (314) | 68.2% (214/314) | 100% (314/314) | 31.5% (99/314) | 30.3% (95/314) |
| Oxidation-reduction process (187) | 40.6% (76/187) | 52.9% (99/187) | 100% (187/187) | 64.2% (120/187) |
| Oxidoreductase activity (157) | 47.1% (74/157) | 60.5% (95/157) | 76.4% (120/157) | 100% (157/157) |
| Fatty acid biosynthetic process (14) | 14.3% (2/14) | 21.4% (3/14) | 78.6% (11/14) | 71.4% (10/14) |
| Iron ion binding (50) | 12% (6/50) | 4% (2/50) | 72% (36/50) | 34% (17/50) |
|  | **AATMA/AAT*** | **Transport** | **Transporter activity** | **Transmembrane Transport** |
| AATMA/AAT (18) | 100% (18/18) | 94.4% (17/18) | 100% (18/18) | 100% (18/18) |
| Transport (80) | 21.3% (17/80) | 100% (80/80) | 38.8% (31/80) | 83.8% (67/80) |
| Transporter activity (33) | 0.0% (0/33) | 93.9% (31/33) | 100% (33/33) | 87.9% (29/33) |
| Transmembrane transport (161) | 11.2% (18/161) | 41.6% (67/161) | 18.0% (29/161) | 100% (161/161) |
|  | **CMP** | **Hydrolase activity-OGC** |  |  |
| CMP (45) | 100% (45/45) | 64.4% (29/45) |  |  |
| Hydrolase activity-OGC (29) | 100% (29/29) | 100% (29/29) |  |  |

AATMA is amino acid transporter activity; AAT is amino acid transport; CMP is carbohydrate metabolic process; OGC is O-glycosyl compounds

*Differentially expressed genes for AATMA and AAT were the same (100% overlap for both ontologies). The number of DE genes in the gene ontology column is listed in parentheses.
