# Supplementary figures and images for: Fungal Morphology, Iron Homeostasis, and Lipid Metabolism Regulated by a GATA Transcription Factor in Blastomyces dermatitidis
Source: PLoS Pathog. 2015 Jun 26;11(6):e1004959. doi: 10.1371/journal.ppat.1004959 (PMC4482641; doi:10.1371/journal.ppat.1004959)

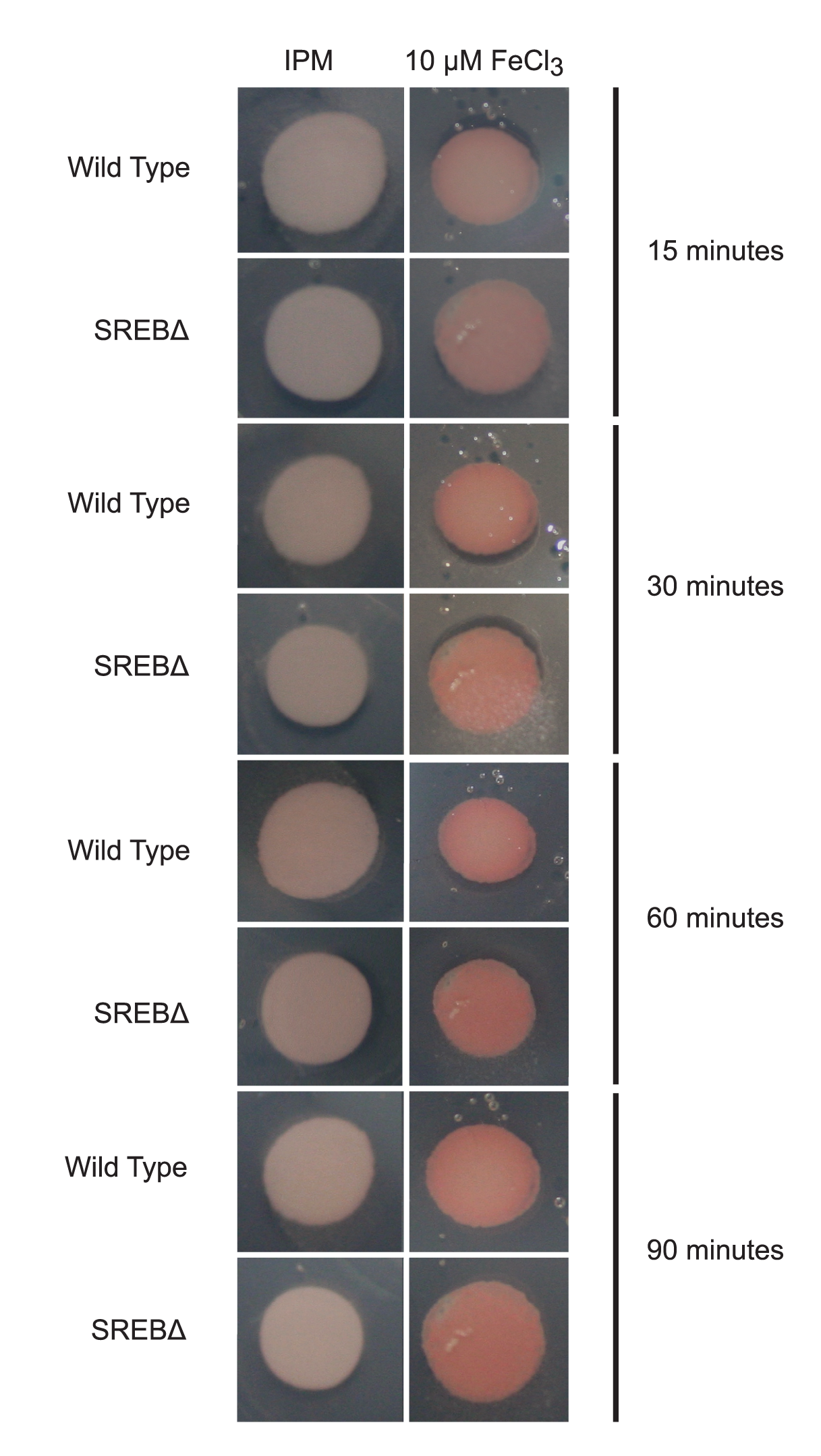

Supplement: S4 Fig — Wild type and SREB∆ yeast (5 x 10^5^ cells) grown on iron poor HMM (IPM) and iron-replete (10 μM FeCl3) media were exposed to 0.1% triphenyltetrazolium chloride (TTC) for 15, 30, 60, and 90 minutes. Reduction of TTC results in the production of red pigmentation (formazan). (TIF) [file ppat.1004959.s011.tif]

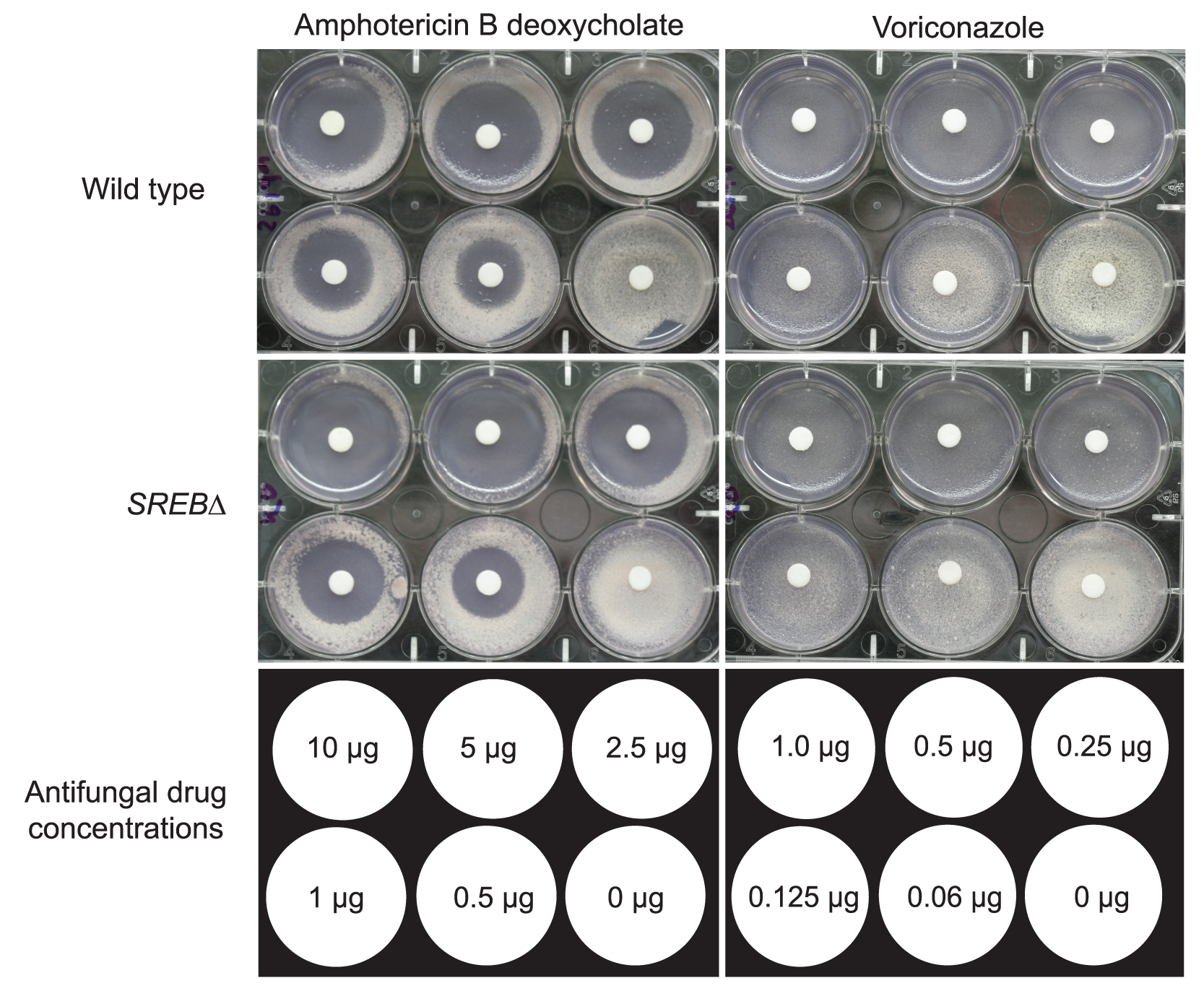

Supplement: S6 Fig — Disc diffusion testing for amphotericin B deoxycholate (0–10 μg) and voriconazole (0–1 μg). (TIF) [file ppat.1004959.s013.tif]

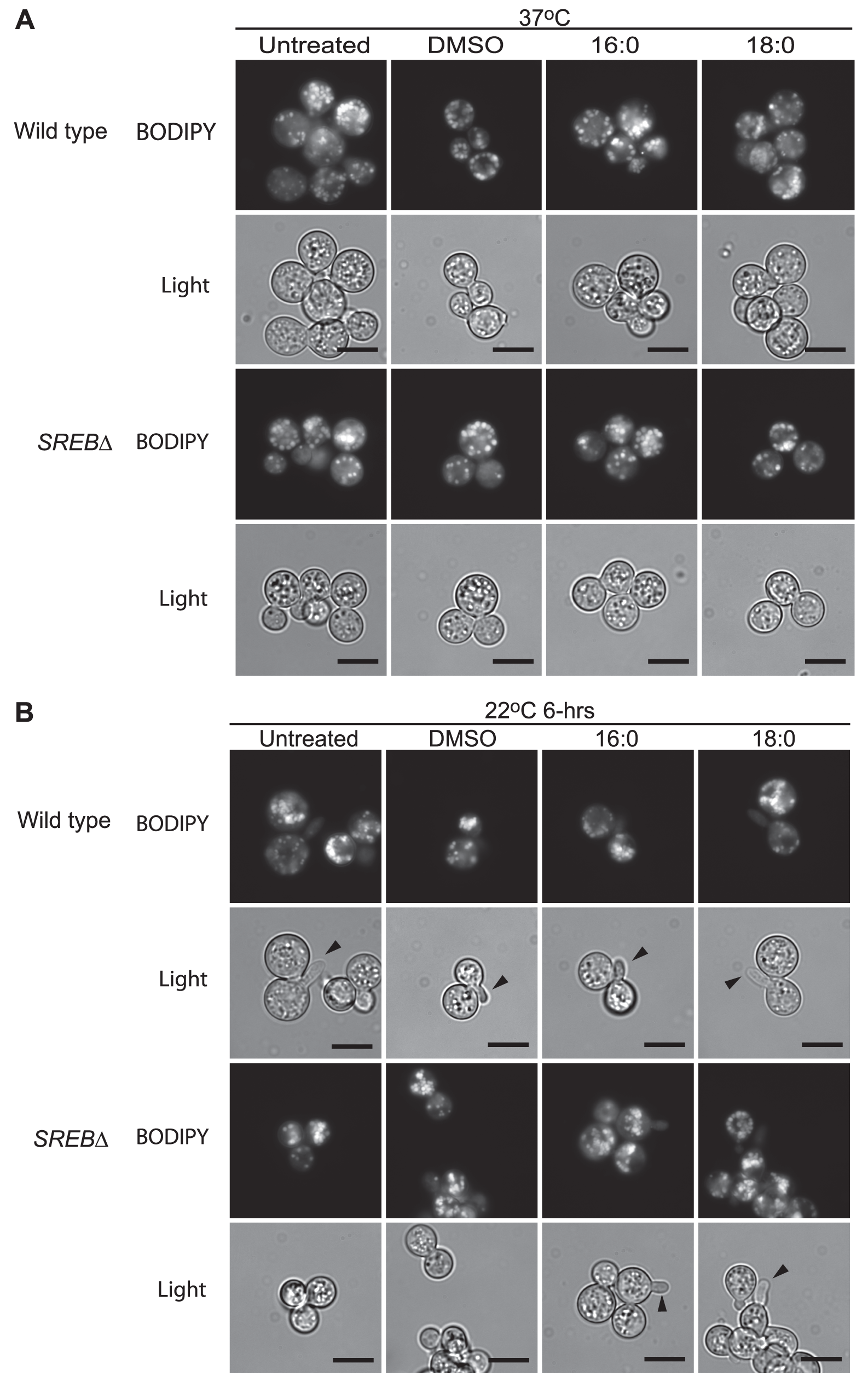

Supplement: S7 Fig — (A) Morphology and lipid droplet straining (BODIPY 493/503) of wild type and SREB∆ cells treated with 0.5 mM palmitic (16:0) and stearic (18:0) acid at 37°C. Controls included untreated cells and cells grown in DMSO. Corresponding bright field microscopic images (light) are below the fluorescent images. Scale bar equals 10 μm. (B) Morphology and lipid droplet straining (BODIPY 493/503) of wild type and SREB∆ cells treated with 0.5 mM palmitic (16:0) and stearic (18:0) acid at 6-hrs 22°C. Controls included untreated cells and cells grown in DMSO. Corresponding bright field microscopic images (light) are below the fluorescent images. Black arrows indicate germ tubes. Scale bar equals 10 μm. (TIF) [file ppat.1004959.s014.tif]

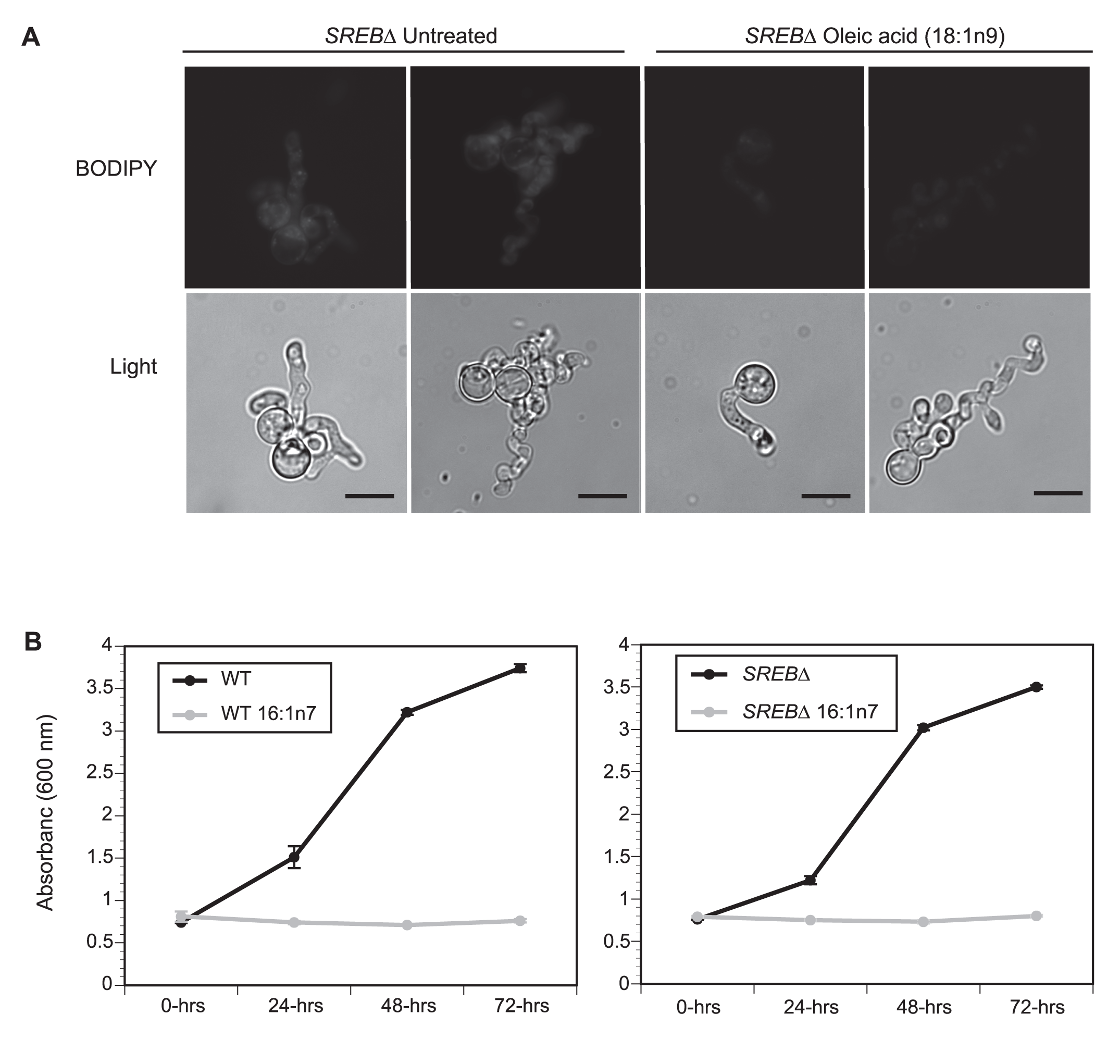

Supplement: S8 Fig — (A) Morphology and lipid droplet staining of SREB∆ untreated and SREB∆ 18:1n9 treated cells at 48-hrs 22°C. (B) WT and SREB∆ cells grown in iron-replete liquid HMM with or without supplementation of 0.125 mM palmitoleic acid (16:1n7) at 37°C. Growth was measured by absorbance at 600 nm. Results are averaged from 2 biological replicates from a representative experiment. (TIF) [file ppat.1004959.s015.tif]

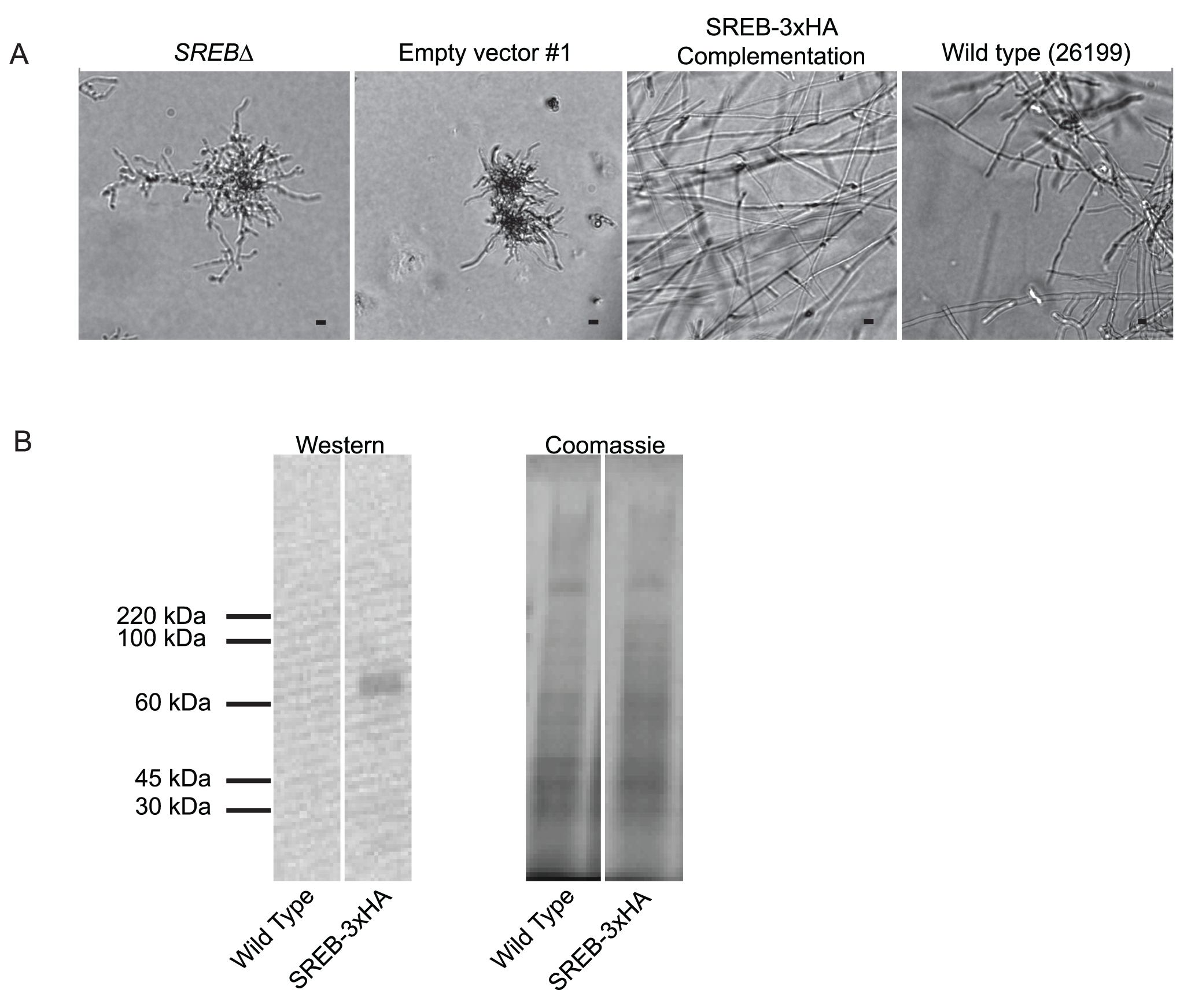

Supplement: S9 Fig — (A) The morphologic defect of SREB∆ at 22°C was successfully complemented by the SREB-3xHA construct. SREB∆, empty vector, complemented strains, and wild type strains were imaged at 17 days 22°C. Scale bar is equals 10 μM. (B) Western blot and coomassie gel analyses of wild type 26199 and 26199 transformed with SREB-3xHA. Predicted molecular weight of SREB-3xHA was 71.2 kDa. (TIF) [file ppat.1004959.s016.tif]
